# In silico protein engineering shows that novel mutations affecting NAD^+^ binding sites may improve phosphite dehydrogenase stability and activity

**DOI:** 10.1038/s41598-023-28246-3

**Published:** 2023-02-01

**Authors:** Soukayna Baammi, Rachid Daoud, Achraf El Allali

**Affiliations:** African Genome Centre (AGC), Mohammed VI Polytechnic University, Benguerir, Morocco

**Keywords:** Molecular modelling, Protein design

## Abstract

*Pseudomonas stutzeri* phosphite dehydrogenase (PTDH) catalyzes the oxidation of phosphite to phosphate in the presence of NAD, resulting in the formation of NADH. The regeneration of NADH by PTDH is greater than any other enzyme due to the substantial change in the free energy of reaction (G°′ = − 63.3 kJ/mol). Presently, improving the stability of PTDH is for a great importance to ensure an economically viable reaction process to produce phosphite as a byproduct for agronomic applications. The binding site of NAD^+^ with PTDH includes thirty-four residues; eight of which have been previously mutated and characterized for their roles in catalysis. In the present study, the unexplored twenty-six key residues involved in the binding of NAD^+^ were subjected to in silico mutagenesis based on the physicochemical properties of the amino acids. The effects of these mutations on the structure, stability, activity, and interaction of PTDH with NAD^+^ were investigated using molecular docking, molecular dynamics simulations, free energy calculations, and secondary structure analysis. We identified seven novel mutations, A155I, G157I, L217I, P235A, V262I, I293A, and I293L, that reduce the compactness of the protein while improving PTDH stability and binding to NAD^+^.

## Introduction

Phosphorus is essential for the survival of all life forms. It is essential for processes such as cellular metabolism, energy production, and membrane integrity, as well as for the storage and transmission of genetic information^1,2^. Therefore, phosphorus uptake is an essential process for all organisms. In nature, phosphorus is almost always present in the form of inorganic phosphate (PO43-), the only form that cells can directly use for biological functions^3^. Although many microbes have mechanisms for uptake of phosphate with high affinity, the lack of phosphate inhibits their growth because the availability of this nutrient varies greatly depending on the habitat^4^. To circumvent this problem, some bacteria can import and metabolize reduced phosphorus molecules such as phosphite (HPO32-), hypophosphite (H2PO2-), and organophosphonates. *Pseudomonas stutzeri WM88*, a Gram-negative soil bacterium, is an example of an organism that can use these molecules as growth-promoting phosphorus sources^5^.

In the presence of NAD^+^, *Pseudomonas stutzeri WM88* phosphite dehydrogenase (PTDH) catalyzes the oxidation of phosphite to phosphate, producing NADH^1^. The *PtxABCD* operon of *P. stutzeri* contains the *Ptx*A, *Ptx*B and *Ptx*C genes, which encode the binding protein-dependent transporter system that controls the use of Phi by the cell. The *PtxD* gene encodes the PTDH enzyme^2^, which is not inhibited by the end products. To date, sulfite is considered the only known inhibitor of the PTDH enzyme^3^.

PTDH has received considerable research attention. It has been investigated as a system for the regeneration of cofactor NADH with a much higher free energy shift during the oxidation of phosphite to phosphate (G°′ = − 63.3 kJ/mol), ensuring an economically viable reaction process^4–6^. PTDH is also been studied as a new selectable marker for the genetic transformation of plants^7^ and for the development of phosphite-based weed control and fertilizer systems^8^. As a result, there is great interest in several research studies to improve PTDH's stability for agronomic applications.

Point mutations in protein structure can lead to novel dynamical features and conformational modifications^9^ that affect protein activity and stability^10^. The mechanism of such alterations behind a mutation is being explored to better understand their effect on PTDH structure, stability, and activity^11,12^. Recently, we investigated how a series of mutations in *P. stutzeri* applied in the laboratory and deposited in the Brenda database (https://www.brenda-enzymes.org/) affected PTDH-NAD^+^ interaction and structural stability^13^. As a result, we found mutations acting on the NAD^+^ binding site, such as the K76 mutants R237K and E266Q, while the others affect the structural stabilization and the activity of PTDH catalysis, or both.

Several previous studies using in silico mutagenesis-based approaches were interested in exploring the thirty-four residues involved in NAD^+^ binding. However, only eight were subject to mutagenesis to characterize their role in catalysis. In the present study, the unexplored twenty-six key residues involved in NAD^+^ binding were subjected to in silico mutagenesis based on the physicochemical properties of amino acids.

Computational techniques such as molecular docking and molecular dynamics simulations are used to identify novel mutations that would improve the stability and activity of PTDH. We have made specific amino acid changes in the NAD^+^ binding residues and tested their effects on the NAD^+^ interaction. The structural characteristics and stability of the mutants that improved the interaction were then assessed to better understand the relationship between structure and function. Surprisingly, we found that A155I, G157I, L217I, P235A, V262I, I293A, and I293L increased the protein's stability and affinity for NAD^+^.

## Material and methods

The 3D structure of PTDH (accession ID: 4E5N) was retrieved from the PDB databank^14^. The water molecules were eliminated from the protein file before starting downstream analyses^15^. Chimera v1.14 was used to inspect the structure for any fractures or missing residues. The structure was then subjected to protonation state correction^16^. Chain A has been chosen as a template for in silico mutagenesis studies.

### Preparation of the in-silico mutants

The binding site of NAD^+^ comprises thirty-four residues; eight of which have been investigated in previous studies for their roles in catalysis using mutagenesis. Therefore, we focused on the remaining twenty-six amino acids, namely Gly77, Pro97, Leu100, Thr104, Leu151, Gly152, Met153, GLy154, Ala155, Ile156, Gly157, His174, Lys177, Ala207, Leu208, Pro209, Leu210, Asn211, Thr214, Leu217, Pro235, Cys236, Asp261, Val262, Ile293, and Gly294. Each residue in the binding site was replaced by another residue with the same physicochemical character using Chimera’s mutagenesis module with the default parameter^17^. After the introduction of each mutation, the corresponding proteins were subjected to energy minimization to remove incorrect atom molecular geometries. These mutated structures were used for further analysis.

### Molecular docking

AutoDock vina software (ADV) was used to study the molecular docking of all mutants with the same ligand (NAD^+^)^18^. The polar hydrogen atoms and Gasteiger charges were added to the protein chain^19^. The ligands were subjected to the same procedure to ensure that the torsions for rotation were accurately adopted during docking^20^. The macromolecules were under rigid conditions when interacting with the ligand under flexible conditions, using the Lamarckian Genetic Algorithm (LGA) method^21^. For both wild type (WT) and mutants, the grid box spacing was set to 0.708 Å, the center was set to (12.378 Å, 6.069 Å, 1.193 Å) and the grid size was set to 20 Å × 20 Å × 20 Å. For each protein–ligand complex, nine poses were constructed based on the docking affinity. The pose with the lowest binding energy and the maximum number of bonds was then chosen for further MD simulation.

### Redocking

To measure docking accuracy, the RMSD of heavy atoms between the docked poses and the crystallographic poses of the ligand (NAD^+^) was calculated^22^. A molecular docking protocol is reliable if the root-mean-square deviation (RMSD) is less than 2 Å^23^.

### Molecular dynamics simulation

Molecular dynamics (MD) simulation is an important tool for determining the affinities of the ligand within the binding site and for investigating the stability of complexes^24^. It shows conformational changes over time and allows us to determine whether the target-ligand complex is stable or not^25^. In the present study, the best scoring docked complexes of the best mutants were included as primary coordinates to check their stability during 100 ns simulation using GROMACS (version 2019.3)^13^ with Charm 27 force field^26^. Each complex was solvated in a dodecahedron box (1.0 nm) with TIP3P water model before neutralization in the system with the counter ions^27^. The protein topologies were created using the HARvard Chemistry macromolecular Mechanics force-field (CHARMm ff), while ligand topologies were generated using SwissParam to obtain force field coordinates. In addition, the steepest descent technique was used for energy minimization with the maximum force (Fmax) set to not exceed 1000 kJ/mol/nm. To equilibrate the system at 300 Kelvin and 1 bar pressure, two successive 1 ns simulations were used using canonical NVT and isobaric NPT. The temperature was controlled using the Berendsen thermostat, while the pressure was controlled using the Parrinello–Rahman barostat^28^. MD simulations were performed for 100 ns in 50,000,000 steps storing the coordinate data for every 2 fs. All preliminary analyses, including root mean square deviation (RMSD), radius of gyration (Rg), root mean square fluctuations (RMSF), solvent-accessible surface area (SASA), hydrogen bond (H-bond), and principal component analysis (PCA), were carried out using GROMACS.

### Binding energy calculation by MM-PBSA

To calculate binding free energies of the screened complexes, the Molecular mechanics Poisson–Boltzmann surface area (MM-PBSA) was used^29^. The binding free energy (E _Binding_) is obtained by the following equations:

1 $$\Delta {\text{EMMPBSA}} = {\text{ E}}_{{{\text{complex}}}} - \, \left( {{\text{E}}_{{{\text{protein}}}} + {\text{ E}}_{{{\text{ligand}}}} } \right)$$

Equation (1) is the total MMPBSA energy of the protein–ligand complex, E_protein_ and E_ligand_ are the isolated proteins' and ligands' total free energies in solution, respectively.

2 $$\Delta {\text{GMMPBSA}} = \Delta {\text{E}}_{{{\text{vdw}}}} + \Delta {\text{E}}_{{{\text{Elec}}}} + \Delta {\text{E}}_{{{\text{polar}}}}$$

Equation (2) MM-PBSA is the sum of the following energies: electrostatic (E_Elec_), van der Waals (E_vdw_), polar (E_polar_), and nonpolar (E_Apolar_).

### Pathogenicity prediction

Numerous algorithms have been developed to predict whether a mutation is pathogenic or not. We applied the MP3 algorithm, which uses a combination of Support Vector Machine (SVM) and Hidden Markov Model (HMM) to predict the pathogenicity of a protein. To check whether one of the mutations we introduced is pathogenic, we ran the MP3 algorithm through the web server “http://metagenomics.iiserb.ac.in/mp3/index.php” by submitting the fasta file of each protein and keeping the default parameters^30^.

## Result and discussion

### Evaluation of interaction between PTDH and NAD^+^

AutoDock Vina results show the interaction modes and binding parameters for the optimal ligand configuration, including binding energy, H-bonding, hydrophobic forces, and electrostatic interactions, along with associated scores and functions^31^. The critical condition in such interaction is that the ligand should be properly oriented and conformed to fit into the enzyme binding site and form a protein–ligand complex^32^. Therefore, the best docking score, the number of formed hydrogen bonds, and the optimal interactions with the amino acid residues of PTDH were calculated and used as criteria to evaluate the best score among the nine poses obtained by Autodock Vina (Table 1)^32^

**Table 1:** The binding affinity of NAD^+^ with the WT and selected mutants of PTDH.

Enzyme	Affinity (Kcal/mol)	No. of H-bond interactions	Residues involved in hydrogen bonding
WT	− 10.6	6	D261-P235-A155-I156-2K76
C236A	− 11	8	D261-P235-A155-I156-2K76-E175-A175
C236M	− 11	8	D261-P235-A155-I156-2K76-E175-R237
C236S	− 11	7	D261-P235-A155-I156-2K76-A176
C236T	− 11.1	8	D261-P235-A155-I156-2K76-E175-A176
**I293A**	− **11.3**	**10**	**D261-P235-A155-I156-2K76-E175-T104-R237-A176**
I293G	− 11	7	D261-P235-A155-I156-2K76-A176
**I293L**	− **11.3**	**10**	**D261-P235-A155-I156-2K76-E175-T104-R237-A176**
I293V	− 11.1	8	D261-P235-A155-I156-2K76-E175-T104
K177A	− 11.1	8	D261-P235-A155-I156-2K76-E175-A176
K177H	− 11.1	8	D261-P235-A155-I156-2K76-E175-A176
K177R	− 11	7	D261-P235-A155-I156-2K76-A176
L217A	− 11	7	D261-P235-A155-I156-2K76-E175
**L217I**	− **11.2**	**9**	**D261-P235-A155-I156-2K76-E175-R237-A176**
M153A	− 11	7	D261-P235-A155-I156-2K76-A176
**P235A**	− **11.3**	**10**	**D261-A235-A155-I156-2K76-G294-A176-E175-C236**
V262A	− 11	8	D261-P235-A155-I156-2K76-E175-A176
**V262I**	− **11.3**	**9**	**D261-P235-A155-I156-2K76-E175-R237-A176**
V262L	− 11.1	8	D261-P235-A155-I156-2K76-E175-A176
A207G	− 11	6	D261-P235-A155-I156-2K76
A207I	− 11.1	8	D261-P235-A155-I156-2K76-E175-A176
A207L	− 11.1	7	D261-P235-A155-I156-2K76-A176
A207V	− 11	7	D261-P235-A155-I156-2K76-A176
M153C	− 11	8	D261-P235-A155-I156-2K76-E175-A176
M153S	− 11.1	6	D261-P235-A155-I156-2K76
N211A	− 11	8	D261-P235-A155-I156-2K76-E175-A176
N211D	− 11.1	7	D261-P235-A155-I156-2K76-A176
T104A	− 11	7	D261-P235-A155-I156-2K76-A176
**A155I**	− **11.2**	**9**	**D261-P235**-**I155-I156-2K76-L210**-**T104**
G152A	− 11.1	8	D261-P235-A155-I156-2K76-E175-A176
G157A	− 11.1	7	D261-P235-A155-I156-2K76-A176
**G157I**	− **11.3**	**9**	**D261-P235-A155-I156-2K76-E175-R237-A176**
G157L	− 11.1	7	D261-P235-A155-I156-2K76-A176
G157V	− 11.1	7	D261-P235-A155-I156-2K76-A176
L151A	− 11	8	D261-P235-A155-I156-2K76-E175-A176
L151I	− 11	8	D261-P235-A155-I156-2K76-E175-A176

The mutant in bold are selected for MD simulations.

**Figure 1:**
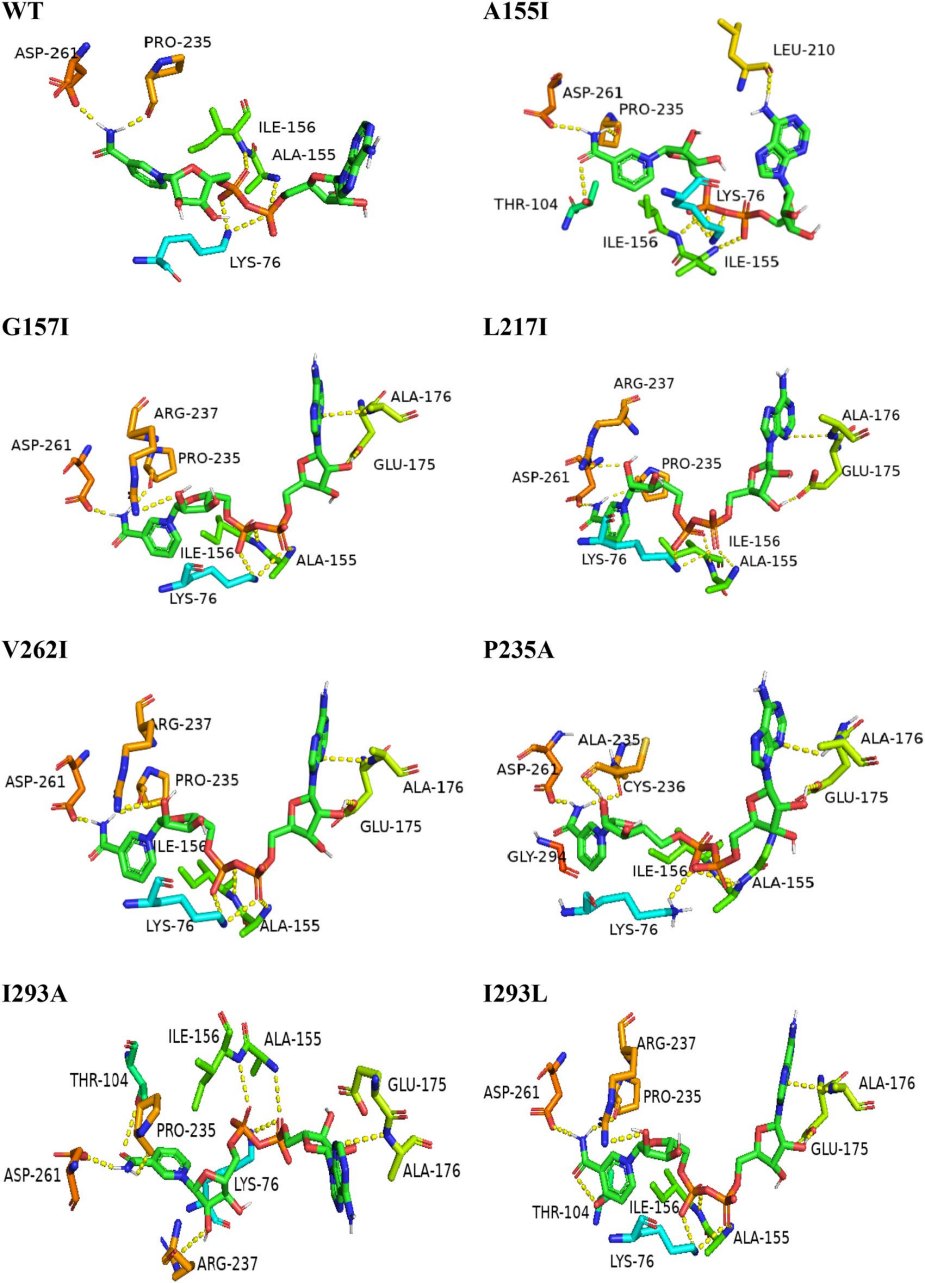
Docked pose of WT and selected mutants PTDH-NAD^+^ based on docking score.

A comparative docking score analysis of all generated mutants with the ligand was performed to find the mutants with the lowest binding energy compared to the WT. Among the selected mutants, A155I, G157I, L217I, P235A, V26I, I293A, and I293L showed the lowest binding energy compared to the reference (− 10.6 kcal/mol). Their interactions at the binding site of PTDH mutants are recognized by the K76, A155, I156, and D261 forming interactions listed in Table 1. In addition, these interactions were compared with those of WT to understand the mechanism of action. These seven mutants were found to have a higher number of residues interacting via binding of the ligand NAD^+^ than WT, suggesting an enhancement and stabilization of the interaction with NAD^+^ (Fig. 1, Fig. S1). However, the remaining twenty-five mutants have a higher binding energy value than WT, suggesting that these mutations alter the conformation of the active pocket and therefore do not allow proper binding of the NAD^+^ (Table S1).

**Figure 2:**
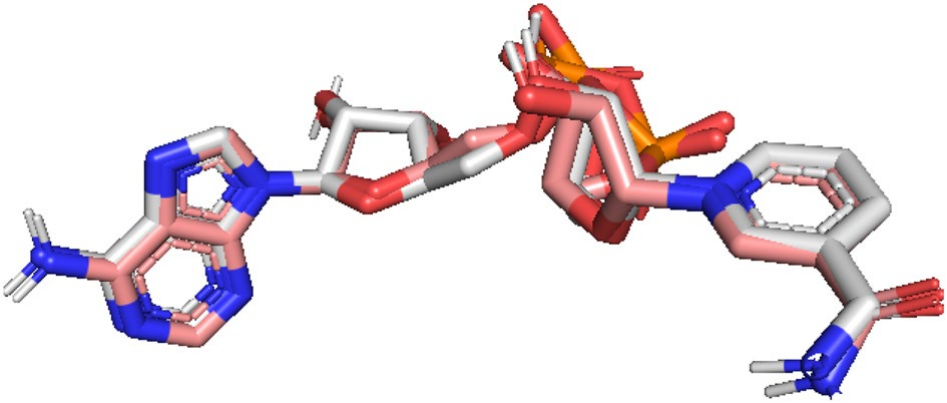
Re-docking pose with an RMSD value of 0.6 Å (white = native ligand, corail = docked ligand).

The RMSD between the original pose and the docked co-crystalline ligand was calculated to validate the docking method, as shown in Fig. 2. The co-crystallized ligand docking result revealed an RMSD value of 0.6, indicating that the approach accurately predicts the binding affinity of the ligand^33^.

### Analysis of pathogenicity variants of PTDH

The prediction of the pathogenicity of the selected mutations concluded that the mutants A155I, G157I, L217I, P235A, V262I, I293A and I293L are non-Pathogenic. L217I, on the other hand, was unclassified.

### Molecular dynamics simulation of protein–ligand complex

**Figure 3:**
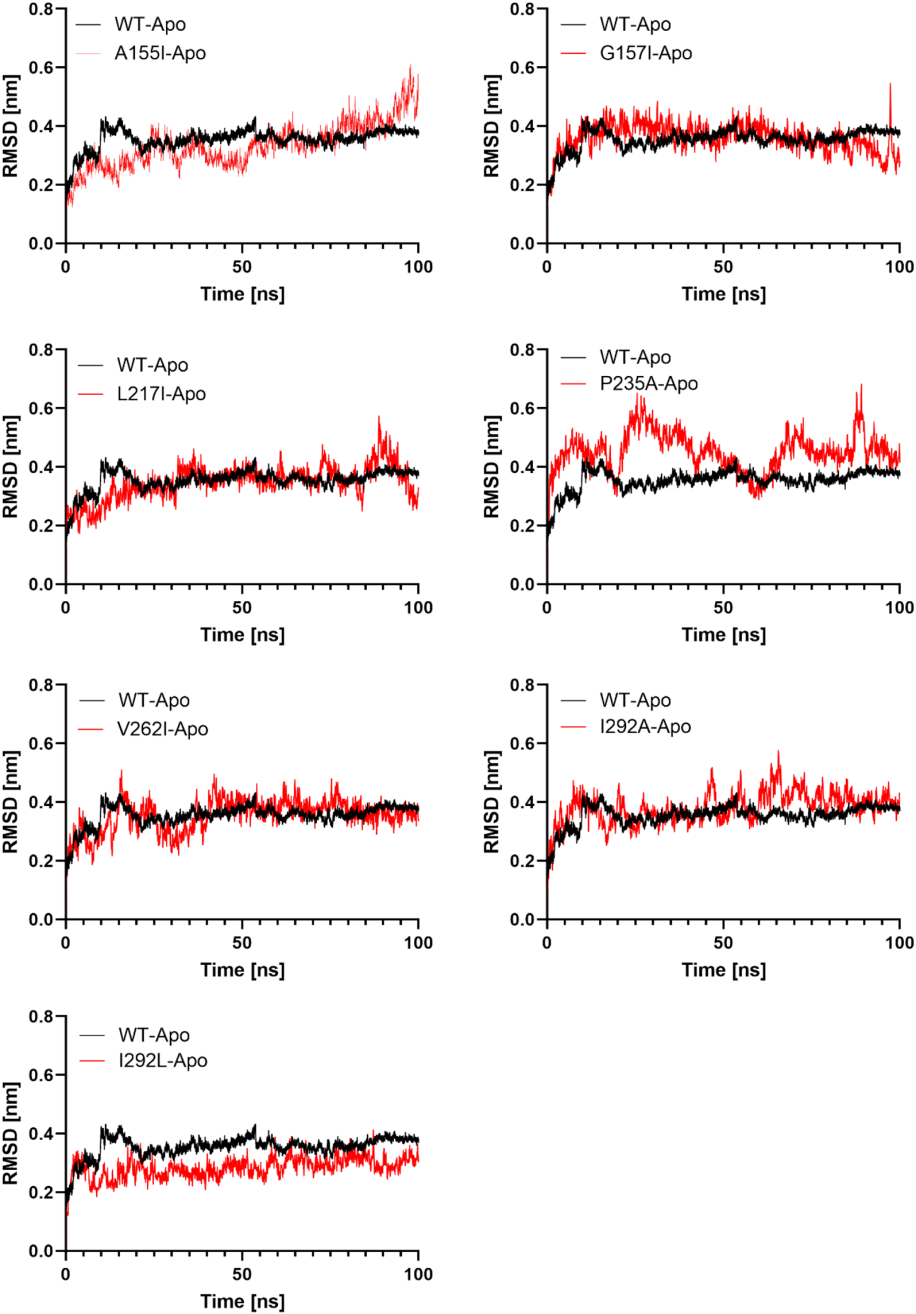
The RMSD of the PTDH WT and mutants in Apo state. The RMSD of the mutant is superimposed on the RMSD of WT. The RMSD in Nanometer is shown on the y-axis, while the simulation time is shown on the x-axis.

MD simulation was used to confirm the stability and logic of the binding mechanisms of the docked complex (protein–ligand)^34,35^. To investigate the effect of mutations on the PTDH-NAD^+^ interaction, the best docked of the WT and seven selected mutants were included as the primary coordinate for MD simulations. The selected mutants, including A155I, G157I, L217I, P235A, V262I, I293A, and I293L, were investigated using 100 ns MD simulation trajectories. Root Mean Square Deviation (RMSD) data were used to assess how the overall protein stability changes with mutation^36^. For all complexes, we calculated the backbone RMSD from the average simulated structure and used it as the primary criterion for measuring the convergence of the system^37^. The backbone RMSD was calculated for the native and mutant structures of the PTDH protein (Fig. 3).

In the apo-state, the RMSD value of WT PTDH reached 0.4 nm between 10 and 20 ns. Thereafter, the RMSD value appeared remain around 0.36 from 25 to 100 ns. For the apo structures A155I and L217I, the RMSD showed a similar profile to WT up to 85 ns, where the RMSD increased to 0.6. In the case of P235A, it appeared to be unstable as it still increased at 100 ns. The RMSD of I293A, I293L, V262I, G157I generally show the same profile as WT. These results suggest that the mutant proteins might have high flexibility in their structure^38,39^ (Fig. 3).

**Figure 4:**
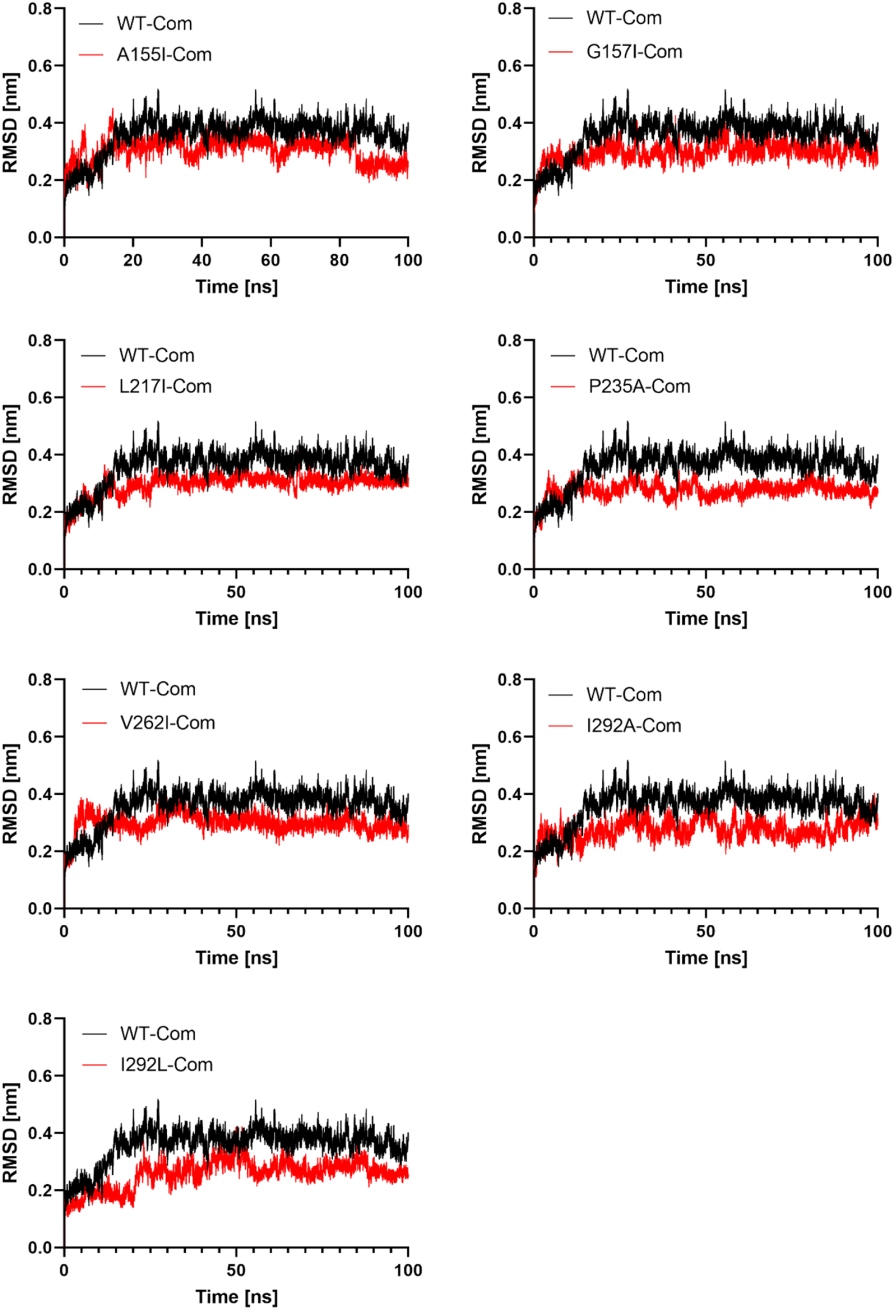
The RMSD of the PTDH WT and complex mutants. The RMSD of the mutant is superimposed on the RMSD of WT. The RMSD in Nanometer is shown on the y-axis, while the simulation time is shown on the x-axis.

In complex with NAD^+^, we found a significant deviation in the structures of the WT in the first 25 ns and the RMSD increased to 0.4 nm. The system then reached equilibrium, and the RMSD remained constant for the next 100 ns. In contrast, the RMSD plots of the seven complex mutants show that they are significantly stable. In the first 20 ns, a slight fluctuation in their RMSD was observed, but without any shift. During the entire simulation time of 100 ns, the RMSD of all systems stabilized and balanced. The backbone RMSD values of the mutants A155I, V262I, I293A, and G157I increased during the initial phase of the simulation. There was a deviation of about 0.3 to 0.4 nm in the RMSD values of the A155I complex between 10 and 18 ns. It then decreased to about 0.3 nm between 20 and 80 ns. The plateau continued and finally settled at 0.25 nm (Fig. 4). The backbone RMSD values of the V262I complex reached 0.4 nm at 15 ns and then dropped to 0.3 nm at 23 ns. The plateau then continued and finally settled at 0.28 nm. Similarly, complexes G157I and I293A reached their backbone RMSD value of 0.29 nm after showing a deflection of 0.31 nm at 11 ns (Fig. 4). The mutants, I293L, P235A, and L217I reached stability at about 20 ns, and no significant deviation of protein backbone atoms was observed for the rest of the simulation time, resulting in a final RMSD value of 0.25 to 0.28 nm. Furthermore, the calculated average RMSDs for all complex mutants ranged from 0.25 ± 0.05 to 0.3 ± 0.04 nm, which is smaller than the average RMSD for WT (0.36 ± 0.06) (Table 3). These results suggest that the replacement of alanine 155, glycine 157, lysine 217, valine 262I with isoleucine increases the number of H-bonding interactions with NAD^+^ as shown in the docking results (Table 1), adopting a stable conformation compared to WT. Replacement of proline 235 (loop-shaped side chain) or isoleucine 293 by alanine stabilizes the mutant complex by engaging the new residues with NAD^+^, suggesting that a simple residue like alanine in these locations increases the stability of PTDH.

### Residual flexibility of wild type and complex mutants

**Figure 5:**
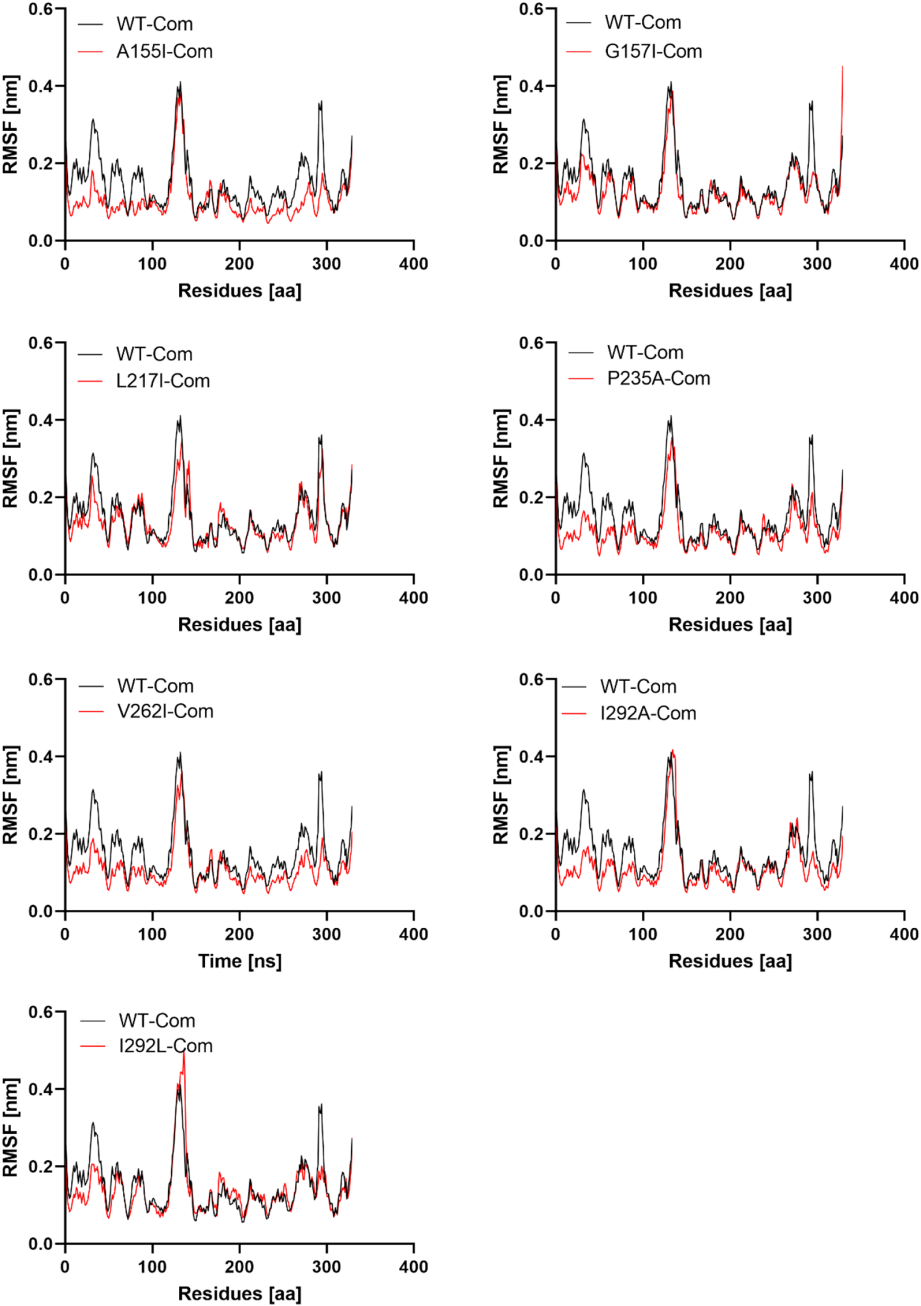
PTDH WT and complex mutant residual flexibility. The x-axis represents the total number of residues, while the y-axis represents the RMSF in nm.

Root Mean Square Fluctuation (RMSF) analysis provides information about the movement of atoms in the flexible protein domains during ligand binding compared to WT^40^. The overall RMSF plots for the WT and the complex mutants with the best docking results are shown in Fig. 5. Compared to the normal position of the residue, higher RMSF values indicate more flexible movements, while low RMSF values indicate limited movements^36^. RMSF plot for WT shows that higher fluctuations are obtained between residues 127–139 and 291–294 with a maximum range of 0.45 nm, indicating lower stability. The same flexibility pattern was reported for mutants I293A and I293L, which showed a fluctuation in regions 135–139 than in the other regions. However, all other mutants complexes showed low fluctuations in all residues of PTDH compared with WT. The number of structural fluctuations after these mutations decreases, which is consistent with the results of the RMSD output. The occurrence of this phenomenon can be associated with the interaction potency of these mutants with NAD^+^ (Table 1). Moreover, the calculated RMSFs average between 0.1 ± 0.04 and 0.13 ± 0.06 nm for all complex mutants, which are smaller than the RMSF average for WT 0.14 ± 0.06 nm (Table 3), suggesting that mutants A155I, G157I, L217I, P235A, V262I, I292A, I293L affected all regions of the protein and reduced the flexibility of PTDH^38^. The effect of the best complex mutants on PTDH folding was tracked by evaluating the Rg functions.

### The structural compactness of WT and the best mutant systems

The gyration radius (Rg) is the mass-weight root mean square distance of a group of atoms from their common center of mass^41^. Rg has been applied to better understand how complexes behave over time in molecular dynamics simulations and how this affects structural compactness^42^. Any ligand with a sufficiently high Rg value is more likely to be flexible, which makes it unstable. Lower Rg values, on the other hand, indicate a conformation that is tightly and densely packed^43^.

**Figure 6:**
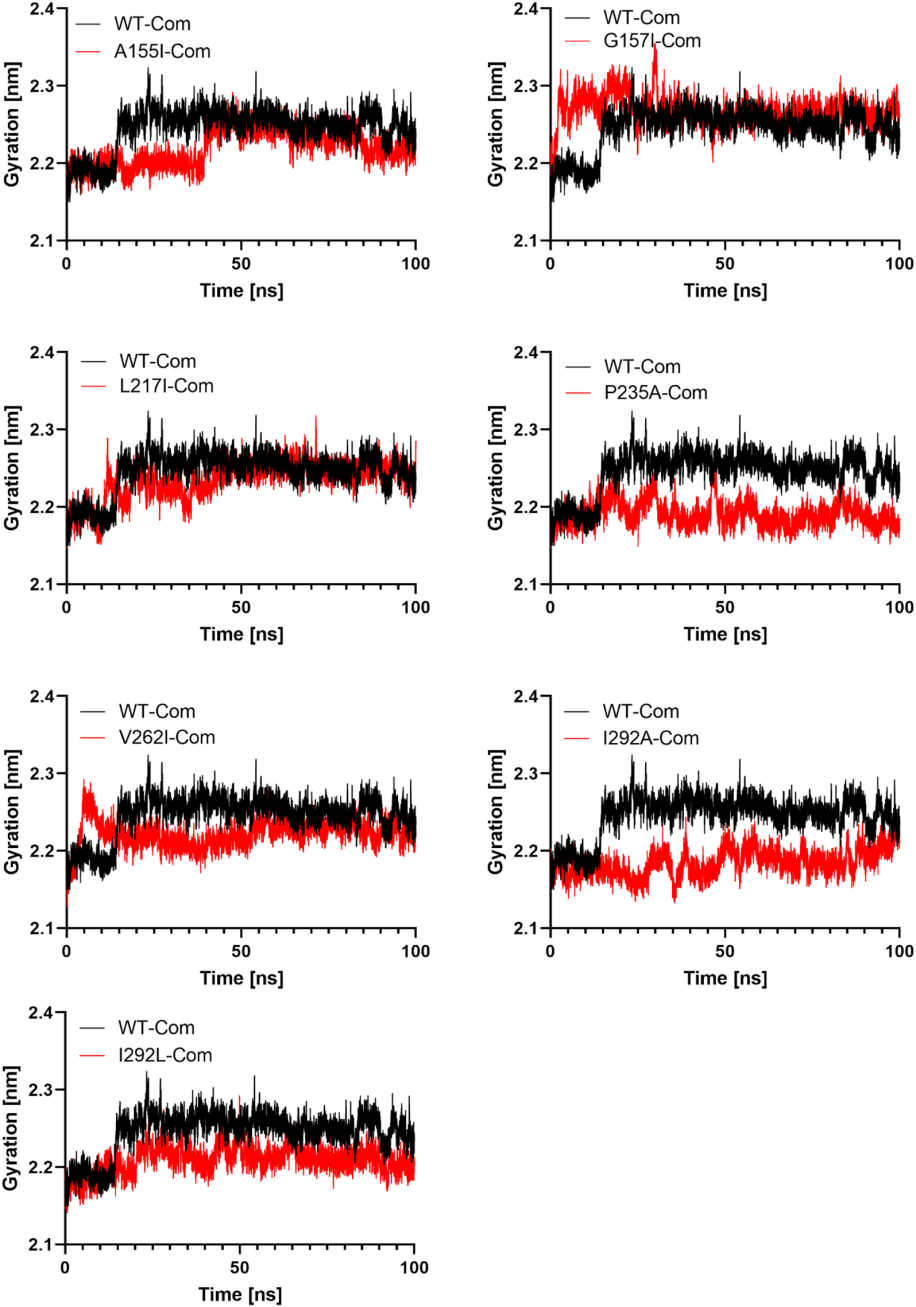
The Rg (gyration radius) of the WT and complex mutants. The x-axis shows the time in nanoseconds, while the y-axis shows the Rg (radius of gyration).

The structural compactness of WT and the best mutant systems is a distinguishing feature that could help us better understand how internal dynamics are affected^28^. The average R_g_ values for mutants A155I, G157I, L217I, P235A, V262I, I293A, and I293L were 2.21, 2.23, 2.23, 2.19, 2.22, 2.18, and 2.20 nm, respectively (Table 3). From the Rg plot, we can see that the WT complex structure has a larger Rg value (2.24) than the complex mutants, making the wild type protein structure relatively unstable compared to the mutants^36^ (Fig. 6).

**Figure 7:**
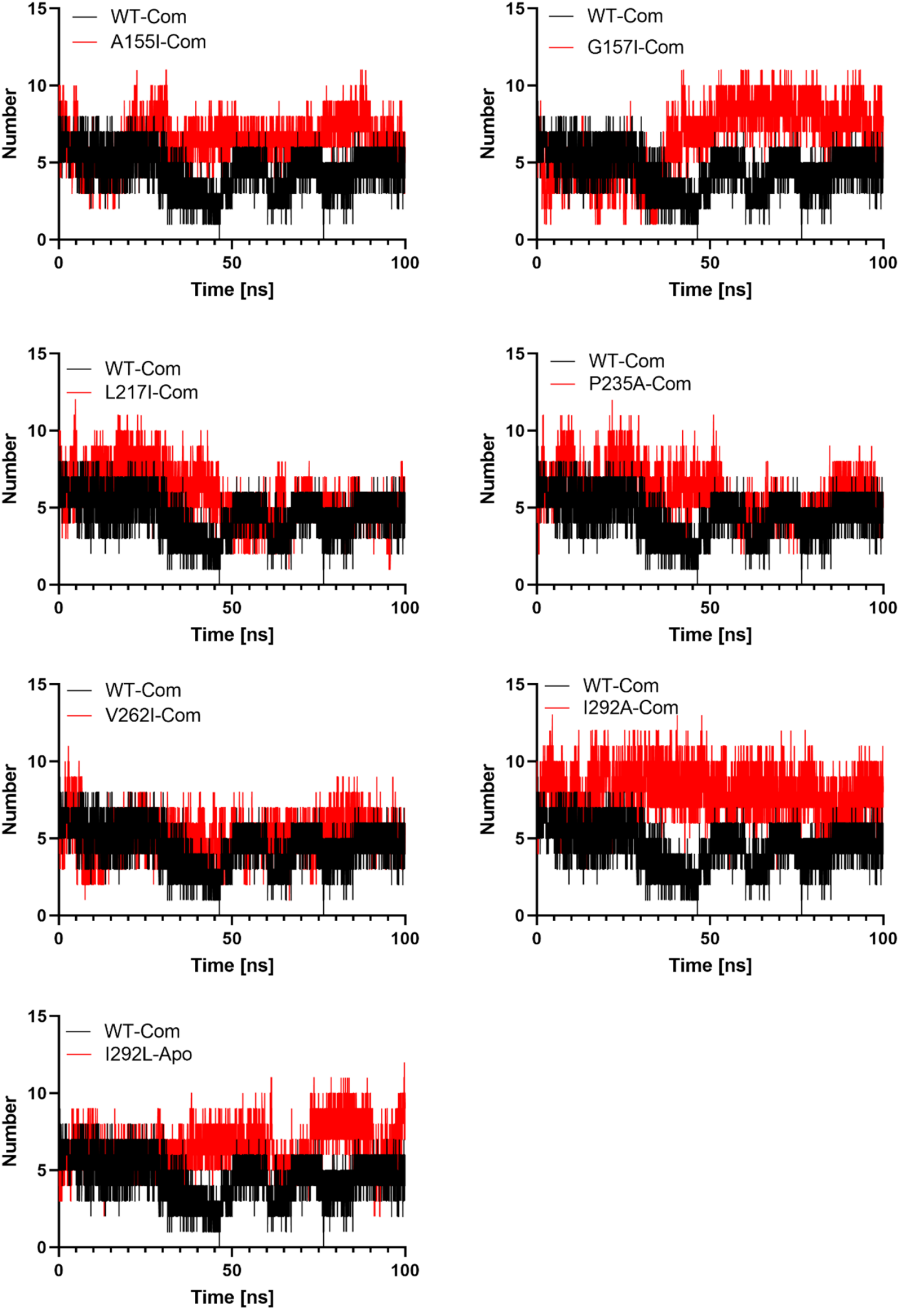
Number of hydrogen bonds between protein and ligand for 100 ns trajectory.

The interactions of hydrogen bonds between the protein PTDH (WT and mutants) and the ligand NAD^+^ were studied using 100 ns molecular dynamics to provide specificity of interaction (Fig. 7), which is a key aspect for molecular recognition^44^.

### Hydrogen bond analysis

In a solvent environment, the hydrogen bonds paired within 0.35 nm between NAD^+^ and PTDH mutants were calculated during MD simulations to verify the stability of the docked complexes^45^. The simulation results showed that the ligand NAD^+^ binds better to the active pocket of mutants A155I, G157I, P235A, I293A, and I293L via hydrogen bonds that average 6.41, 6.49, 5.97, 8.01, and 6.45 H-bonds, respectively. Also, the average number of H-bonds for mutants L217I and V262I is 5.74 and 5.07, respectively, throughout the simulation. These results show that the PTDH mutants have a higher number of H-bonds than the WT (4.50), resulting in higher stability of these complex mutants of NAD^+^ PTDH.

Distances between ligands and PTDH mutants were calculated from MD trajectories. Table 2 gives the average distances over 100 ns and shows that the average distance for A155I, P235A was 0.19 nm in both cases, confirming the docking results showing strong hydrogen bonding between the corresponding residues and NAD + . The remaining mutants have an average distance between 0.27 and 0.30 nm, indicating that the residues can enhance the interaction with NAD + , which is consistent with other studies^46^.

**Table 2:** The average distance between mutated residues and NAD^+^ during 100 ns MD simulation.

Complex	Average distance from NAD + (nm)
A155I	0.19 ± 0.01
G157I	0.28 ± 0.01
L217I	0.30 ± 0.09
P235A	0.19 ± 0.03
V262I	0.28 ± 0.06
I293A	0.27 ± 0.09
I293L	0.27 ± 0.01

### Solvent accessible surface area

**Figure 8:**
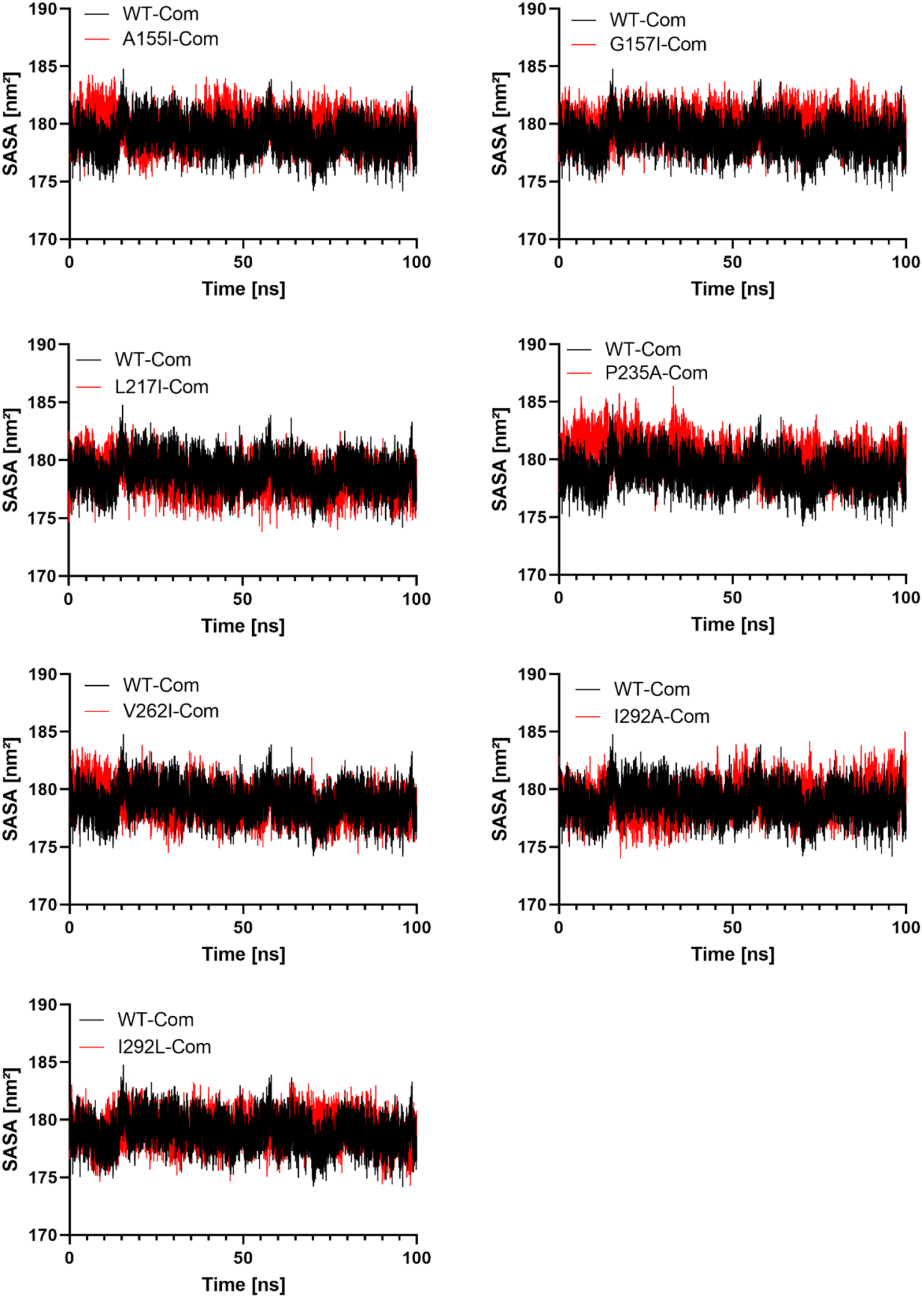
Solvent accessible surface area (SASA) of WT and mutant PTDH protein structures for a 100 ns molecular dynamics simulation. The abscissa is time in (ns), while the ordinate is distance (nm^2^).

Solvent Accessible Surface Area (SASA) is described as the protein surface area in contact with the surrounding solvent^47^. SASA is used to calculate the solvation energy associated with protein binding and structural rearrangement. Explicit solvent models can be used to evaluate these solvation effects. For example, a sphere of water molecules is often used in MD simulations^48^. In the context of available solvent treatments, accessible surface area models (ASA) have become widely used in various applications such as protein MD and structure prediction in general^49,50^. The average SASA values for WT and the mutants A155I, G157I, L217I, P235A, V262I, I293A, and I293L of the PTDH complexes during the 100 ns MD simulations were 178.8, 179.6, 179.8, 178.4, 180.2, 178.8, 179.2, and 179.09 nm^2^ (Table 3). Data analysis showed that there was no significant difference in the SASA values between WT and the mutants of PTDH due to ligand binding (Fig. 8).

**Table 3:** The average RMSD, RMSF and radius of gyration for all complexes, obtained after 100 ns MD simulations.

Complex	Average RMSD (nm)	Average RMSF (nm)	Radius of gyration (nm)
WT	0.36 ± 0.06	0.14 ± 0.06	2.24 ± 0.02
A155I	0.30 ± 0.04	0.10 ± 0.05	2.21 ± 0.02
G157I	0.29 ± 0.03	0.12 ± 0.05	2.23 ± 0.01
L217I	0.29 ± 0.03	0.13 ± 0.05	2.23 ± 0.02
P235A	0.27 ± 0.02	0.11 ± 0.05	2.19 ± 0.01
V262I	0.29 ± 0.03	0.10 ± 0.04	2.22 ± 0.01
I293A	0.27 ± 0.03	0.11 ± 0.06	2.18 ± 0.01
I293L	0.25 ± 0.05	0.13 ± 0.06	2.20 ± 0.01

### Principal component analysis and FEL analysis

**Figure 9:**
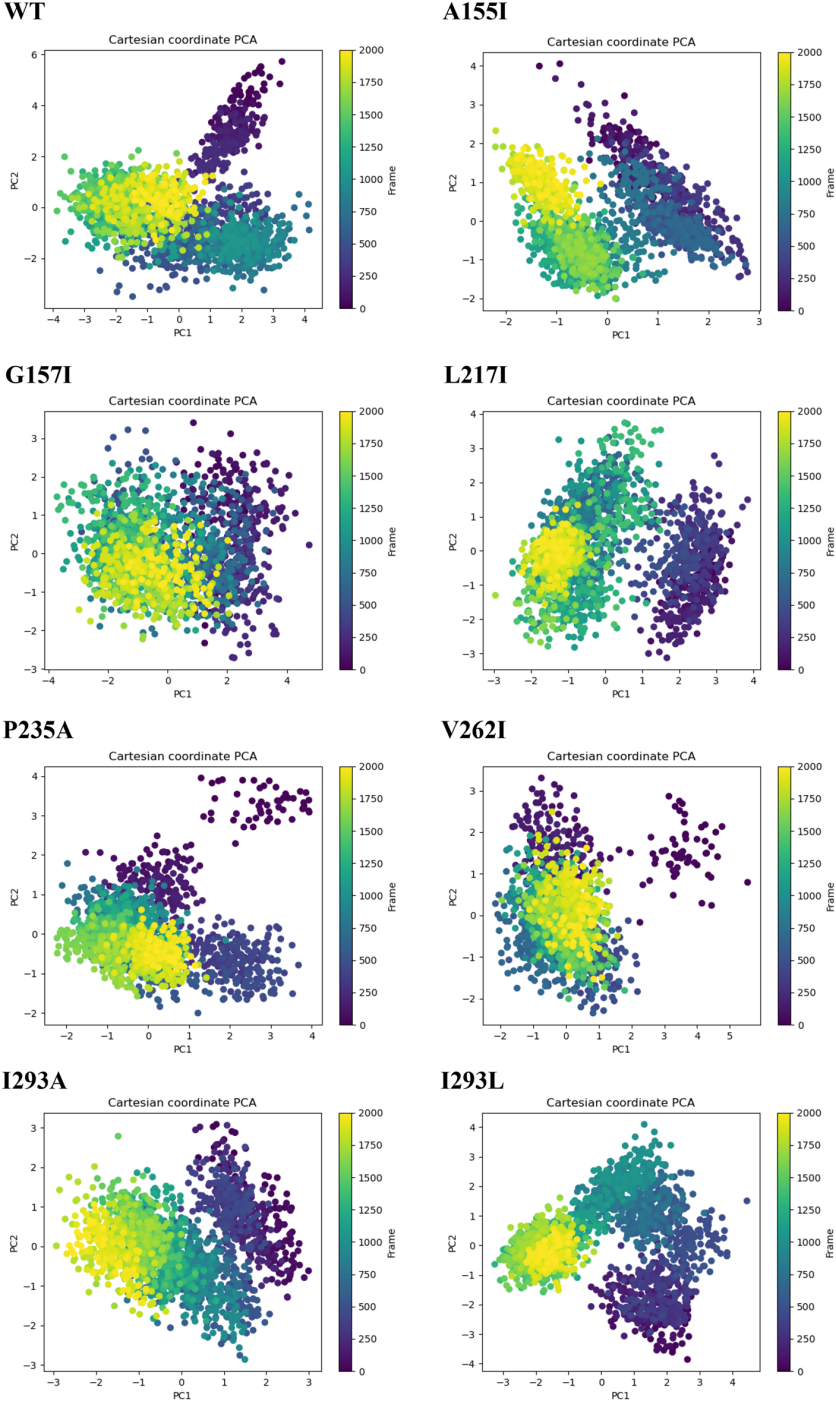
Principal component analysis (PCA) diagram of WT and the mutant PTDH protein structures.

Principal component analysis (PCA) and free energy landscapes (FEL) were extracted to track the effects of the 7 mutations on the energy patterns of the target conformation and the folding of the structures. On the trajectories generated by our simulations, PCA was used to find large-scale collective motions^51^ of the WT and the seven mutants. The projection of PC1 and PC2 from WT and the mutants suggests that A155I, G157I, L217I, V262I, P235A, I293A, and I293L exhibit a more compact type of motion than WT^38^ (Fig. 9).

In the complex with NAD^+^, WT showed cluster motion covering a range on PC1 and PC2 between –4 and 4, and –3 and 6, respectively. However, compared with WT, the PTDH mutants A155I, G157I, L217I, P235A, V262I, I293A, and I293L showed a more compact type of movement on PC1 (between − 2 and 3, − 4 and 4, − 3 and 4, − 2 and 4, − 2 and 5, − 3 and 3, − 3 and 4) and PC2 (− 2 and 4, − 3 and 3, − 3 and 4, − 2 and 4, − 2 and 3, − 3 and 3, − 4 and 4).

**Figure 10:**
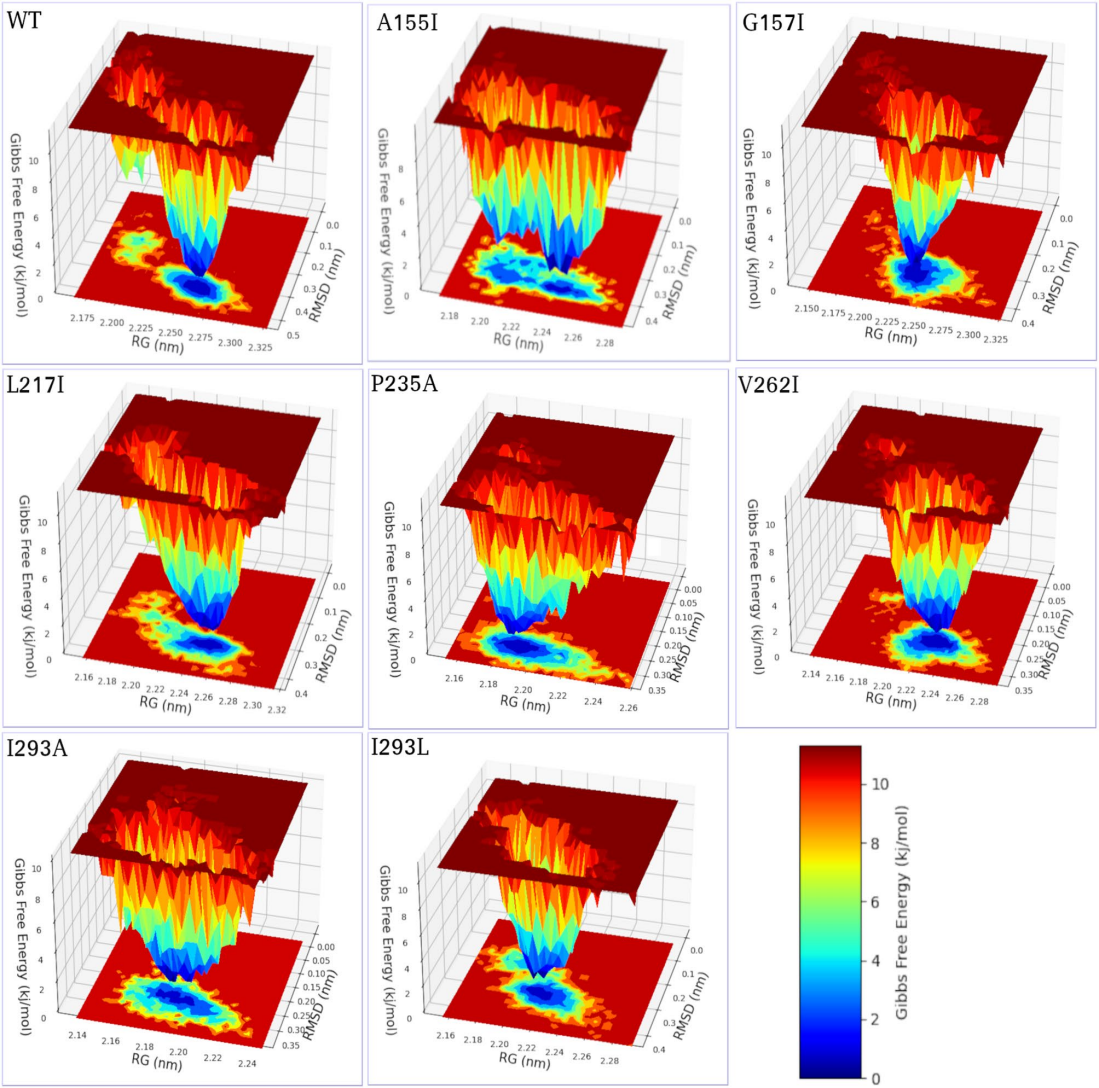
Free energy landscape (FEL) diagram of WT and the mutant PTDH protein structures. Rg, gyration radius; RMSD, root‐mean‐square deviation.

The FEL was built and two reaction coordinates of the free landscape map were defined: one is the RMSD, which indicates the stability of the protein structure, and the other is the gyration radius (Rg), which indicates whether the complex is stably folded. These two reaction coordinates were used to determine the energy landscape. The red color represents a high energy state, while the yellow, green, and blue colors represent the lowest stable states. A higher blue color can be seen in all mutants, indicating that these mutants are more stable than WT. FEL diagrams (Fig. 10) show that the WT achieved the global minima (lowest free energy state) at 0.45 nm and Rg 2. 325 nm. For mutants A155I, G157I, L217I, and I293L, the global minima were reached at 0.4 nm and Rg 2.28, while for mutants P235A, V262I, and I293A, the global minima were reached at 0.35 nm and Rg 2.26. Following these mutations, PTDH folding significantly affects the conformational energy patterns in the complex mutants, which were more concentrated compared to WT. The PCA and FEL results support the RMSD, RMSF, and gyration results, and show that flexibility and structural variation decrease after association of the ligand with these mutations.

### DSSP analysis

To investigate the effects of mutations on the secondary structure of PTDH, an analysis is performed using the Dictionary of Secondary Structure of Protein (DSSP)^52^. Based on the conformational behavior observed in the RMSD, RMSF, and Rg analysis of the WT and mutant PTDH systems, we calculated the content of secondary structure at the mutant position and surrounding residues, which is crucial to detect changes in PTDH structure before and after the induction of site-specific mutations (Fig. S2). As shown in Table 4, we found that the secondary structure components of the adjacent amino acids of PTDH showed an improvement of 2% to 5% when alanine155, leucine217, or valine262 were replaced by isoleucine or proline235 and isoleucine293 by alanine, with the maximum gain (twofold) observed when isoleucine293 was replaced by leucine. However, when glycine157 was replaced by isoleucine in PTDH, the secondary structure components of the surrounding amino acids remained unchanged. It is possible that because of these improvements, PTDH is better able to bind to the cofactor NAD^+^, making it more stable.

**Table 4:** The percentage of secondary structure content for the selected complexes during 100 ns.

Complex mutants	Structure (%)	Coil (%)	Bend (%)	Turn	A-Helix (%)	3-Helix
A155	45	42	12	–	45	–
A155I	49	40	11	4	45	–
G157	64	30	7	–	64	–
G157I	64	31	6	–	63	–
L217	27	50	23	27	–	–
L217I	32	47	21	32	–	–
P235	12	71	16	12	–	–
P235A	15	74	11	15	–	–
V262	2	64	10	2	24	–
V262I	4	63	11	4	22	–
I293	9	74	15	9	–	2
I293A	14	67	17	14	–	2
I293L	18	72	6	18	–	4

### MM-PBSA binding free energy

The package g_mmpbsa was used to determine the average free binding energy of the of WT and selected complexes under GROMACS simulation^53^ (Table 5). The binding energy was calculated by combining the scores of Van der Waal energy, electrostatic energy, polar solvation, and solvent-accessible surface area (SASA) energy. Compared to WT, the total energies of the WT and mutants were (WT/ − 30.277, A155I/ − 51.927, G157I/ − 38.818, L217I/ − 34.943, P235A/ − 43.273, V262I/ − 46.437, I293A/ − 59.176, I293L /− 85.630) indicating that they increase NAD^+^ binding strength. The contributions of VdW, Eletrostatic, and Polar solvation energies to the binding energy of the mutants were higher, suggesting that these mutants have strong NAD binding, which is consistent with the docking results.

**Table 5:** List of observed average and standard deviations of all energetic components including the binding enery taken from MM-PBSA analysis.

Complex mutants	Binding energy (KJ/mol)	SASA energy (KJ/mol)	Polar solvation energy (KJ/mol)	Electrostatic energy (KJ/mol)	Van der Waal energy (KJ/mol)
WT	− 30.277 ± 0.872	− 20.402 ± 0.067	284.696 ± 1.792	− 122.084 ± 1.418	− 172.386 ± 0.840
A155I	− 51.927 ± 0.942	− 23.865 ± 0.059	319.305 ± 1.326	− 141.354 ± 1.028	− 206.059 ± 0.733
G157I	− 38.818 ± 0.713	− 22.448 ± 0.055	259.796 ± 1.170	− 81.712 ± 0.846	− 194.423 ± 0.625
L217I	− 34.943 ± 37.198	− 22.269 ± 1.009	451.645 ± 64.355	− 290.472 ± 48.945	− 173.847 ± 16.793
P235A	− 43.273 ± 1.257	− 16.117 ± 0.095	176.615 ± 2.038	− 76.902 ± 1.070	− 126.847 ± 0.854
V262I	− 46.437 ± 0.840	− 24.238 ± 0.060	356.078 ± 1.661	− 163.231 ± 1.071	− 215.100 ± 0.802
I293A	− 59.176 ± 40.252	− 23.316 ± 1.252	317.898 ± 60.344	− 141.591 ± 39.791	− 212.166 ± 16.943
I293L	− 85.630 ± 1.021	− 28.427 ± 0.044	396.286 ± 1.169	− 184.472 ± 0.643	− 269.063 ± 0.699

In addition, we investigated the contribution of the individual residues of each structure (WT and selected mutants) to the interaction with NAD + in terms of binding free energy. The contribution of each residue was determined by decomposing the total binding free energy of the system into the contributions of each residue (Fig. S3). The results of these analyses show that the replaced residues showed a favorable contribution in the complex mutants, suggesting that these residues may play an important role in the interaction with NAD^+^.

## Conclusion

Phosphite dehydrogenase (PTDH) catalyzes the oxidation of phosphite to phosphate, resulting in the formation of NADH. Although several mutations have been identified in PTDH and attempts have been made to increase the stability of PTDH, the low stability and short half-life of the protein have remained a challenge. In the present study, we used computational techniques including molecular docking, molecular dynamics simulation, and a dictionary of secondary structure protein analysis to discover seven new mutations that improve PTDH stability. Among the in silico generated PTDH mutants, the mutants A155I, G157I, L217I, P235A, V262I, I293A, and I293L were highly capable of enhancing both PTDH stability and activity.

## Supplementary Information

Supplementary Information.

## Data Availability

All available data can be found within the article and in the supplementary file. Further inquiries can be directed to the corresponding author.
