# Supplementary material for: In silico protein engineering shows that novel mutations affecting NAD+ binding sites may improve phosphite dehydrogenase stability and activity
Source: Sci Rep. 2023 Feb 1;13:1878. doi: 10.1038/s41598-023-28246-3 (PMC9892502; doi:10.1038/s41598-023-28246-3)
Supplement: Supplementary file 1 — Supplementary Information. [file 41598_2023_28246_MOESM1_ESM.docx]

# **Improvement of the stability and activity of the phosphite dehydrogenase using in silico protein engineering**

Soukayna Baammi^1^, Rachid Daoud^1*^ and Achraf El Allali^1*^

^1^Mohammed VI Polytechnic University, African Genome Centre (AGC), Benguerir, Morocco

*Corresponding author email: [Rachid.DAOUD@um6p.ma](mailto:Rachid.DAOUD@um6p.ma) , [Achraf.ELALLALI@um6p.ma](mailto:Achraf.ELALLALI@um6p.ma)

Table S1. The binding affinity of NAD^+^ with the WT and mutants of PTDH.

| **Enzyme** | **Affinity (Kcal/mol)** |
| --- | --- |
| WT | -10.6 |
| C236A | -11 |
| C236M | -11 |
| C236S | -11 |
| C236T | -11.1 |
| **I293A** | **-11.3** |
| I293G | -11 |
| **I293L** | **-11.3** |
| I293V | -11.1 |
| K177A | -11.1 |
| K177H | -11.1 |
| K177R | -11 |
| L217A | -11 |
| **L217I** | **-11.2** |
| **l217G** | **-10.4** |
| M153A | -11 |
| **P235A** | **-11.3** |
| V262A | -11 |
| V262G | -10.4 |
| **V262I** | **-11.3** |
| V262L | -11.1 |
| A207G | -11 |
| A207I | -11.1 |
| A207L | -11.1 |
| A207V | -11 |
| M153C | -11 |
| M153S | -11.1 |
| N211A | -11 |
| N211D | -11.1 |
| T104A | -11 |
| **A155I** | **-11.2** |
| A155L | -10.5 |
| A155V | -10.4 |
| G152A | -11.1 |
| G157A | -11.1 |
| **G157I** | **-11.3** |
| G157L | -11.1 |
| G157V | -11.1 |
| L151A | -11 |
| L151I | -11 |
| L210A | -10.4 |
| L210G | -10.5 |
| P209A | -10.3 |
| R137H | -10 |
| R137K | -10.1 |
| L208G | -10.2 |
| L208V | -10.3 |
| L208I | -10.3 |
| D261A | -10.2 |
| D261N | -10.4 |
| G152I | -10.1 |
| G152L | -10 |
| G152V | -10.2 |
| H174A | -10.5 |
| H174R | -9.9 |
| H174K | -10.1 |
| L100A | -10.1 |
| L100G | -10.4 |
| L100I | -10.3 |
| G77V | -10 |
| G77I | -10.3 |

| **A207G**  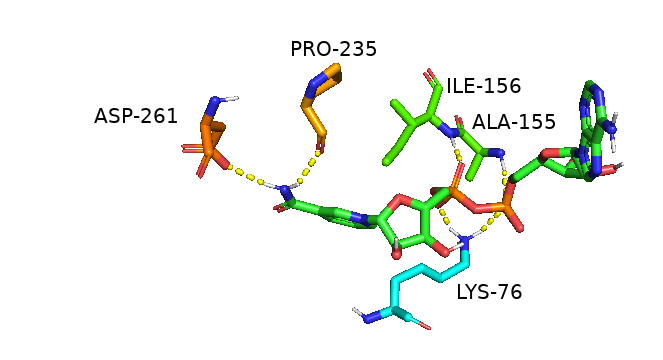 | **A207I**  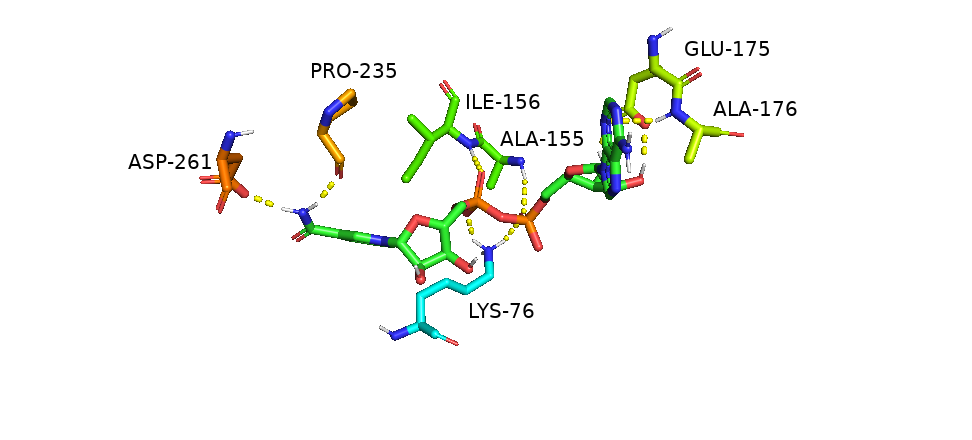 |
| --- | --- |
| **A207L**  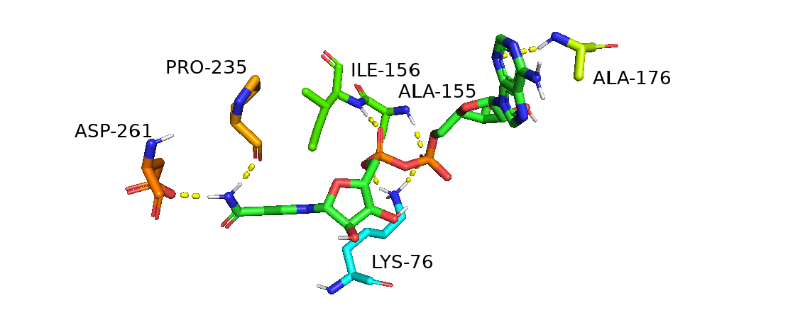 | **A207V**  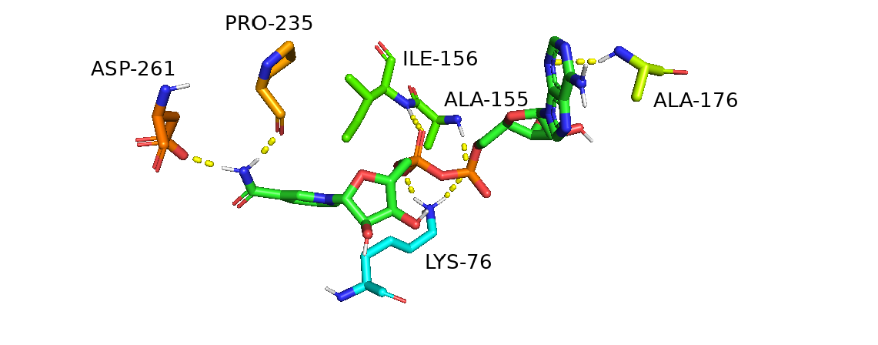 |
| **L217A**  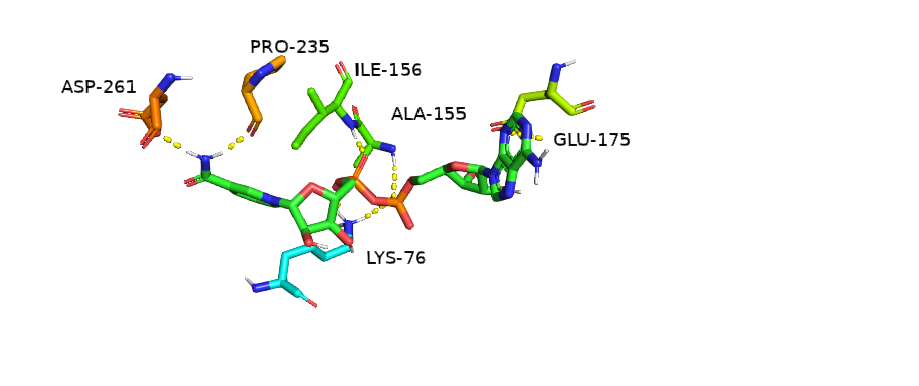 | **T104A**  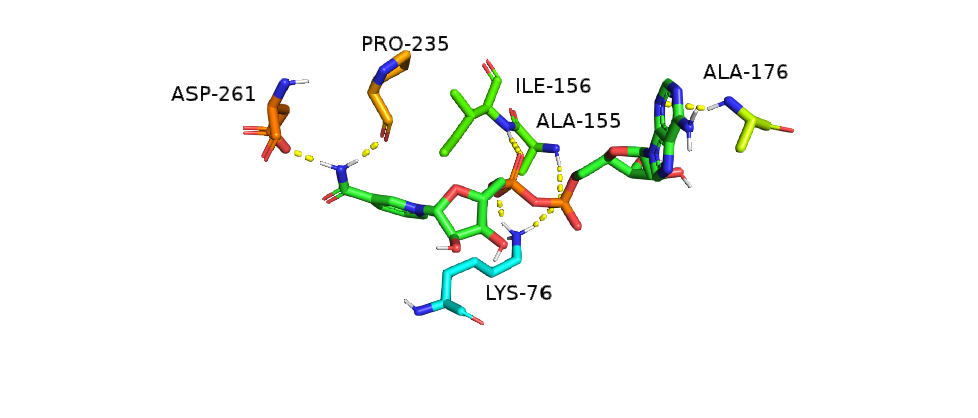 |
| **L151A**  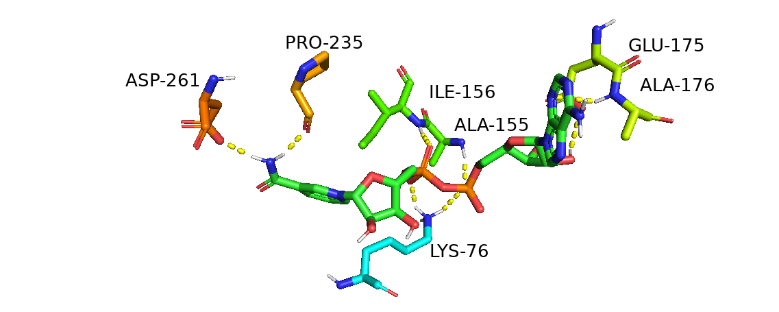 | **L151I**  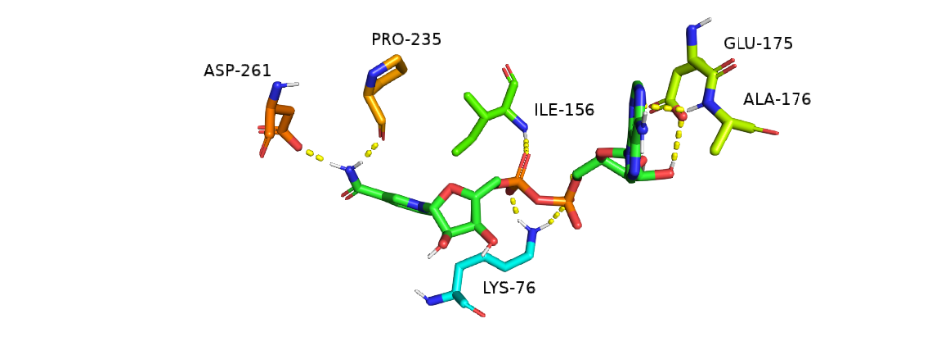 |
| **G152A**  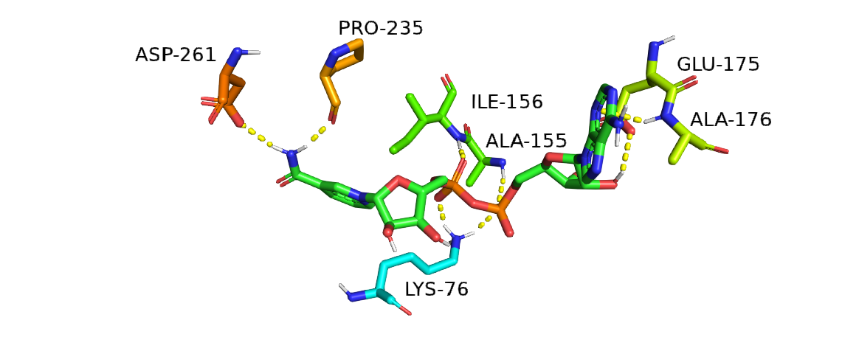 | **M153A**  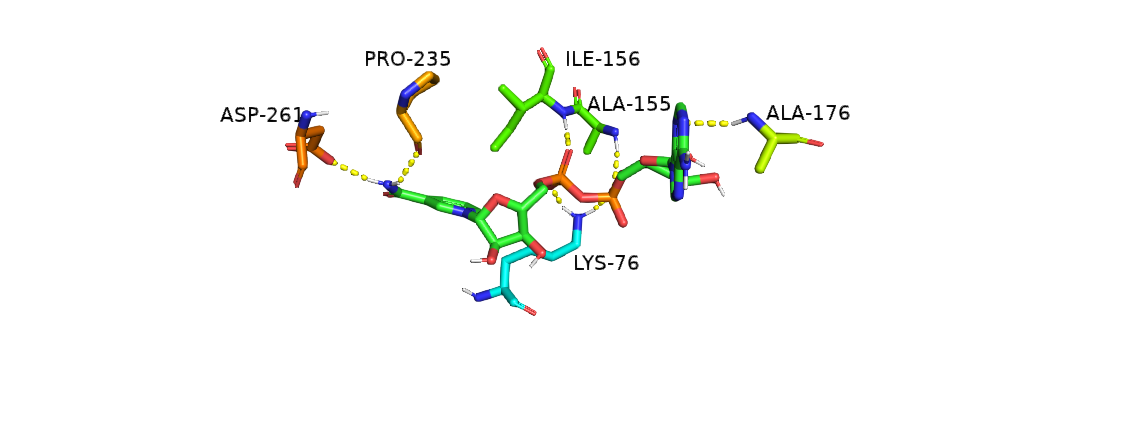 |
| **M153C**  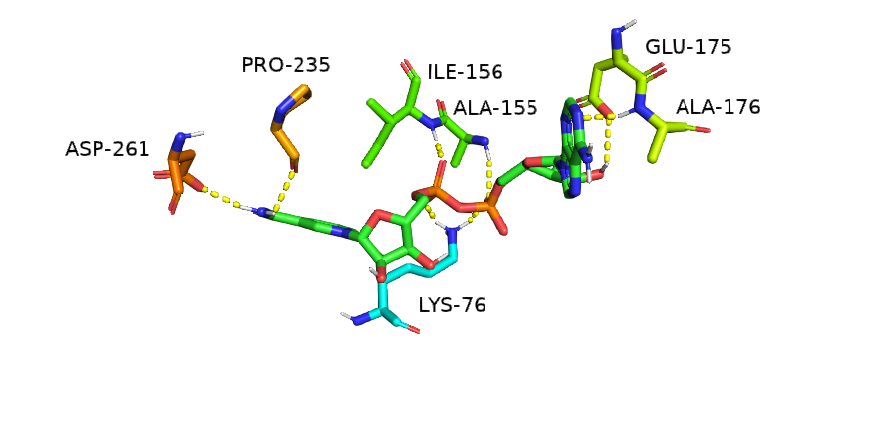 | **M153S**  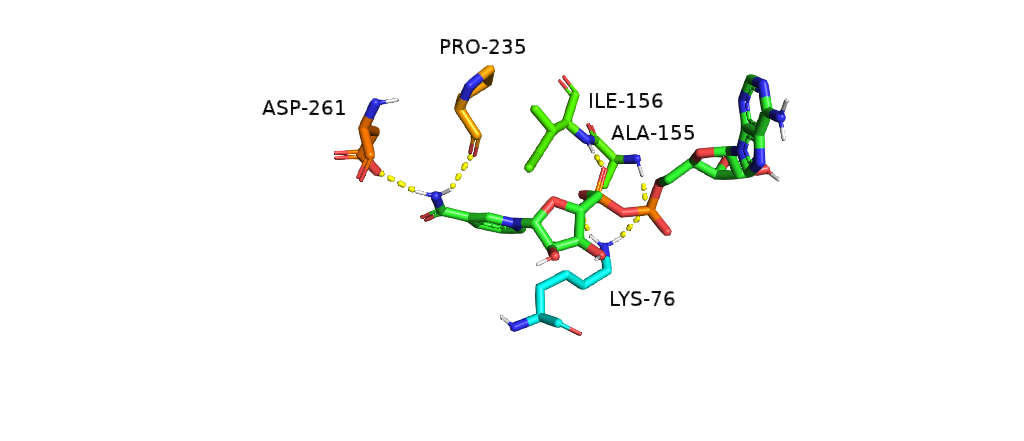 |
| **G157L**  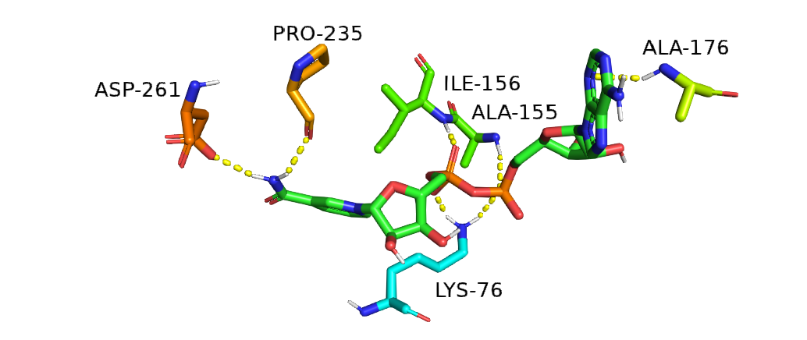 | **G157V**  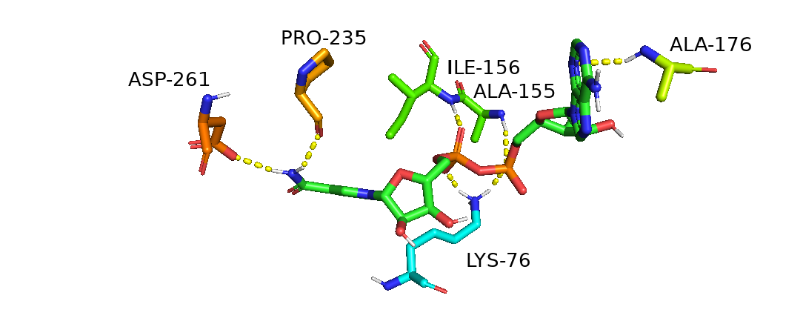 |
| **K177A**  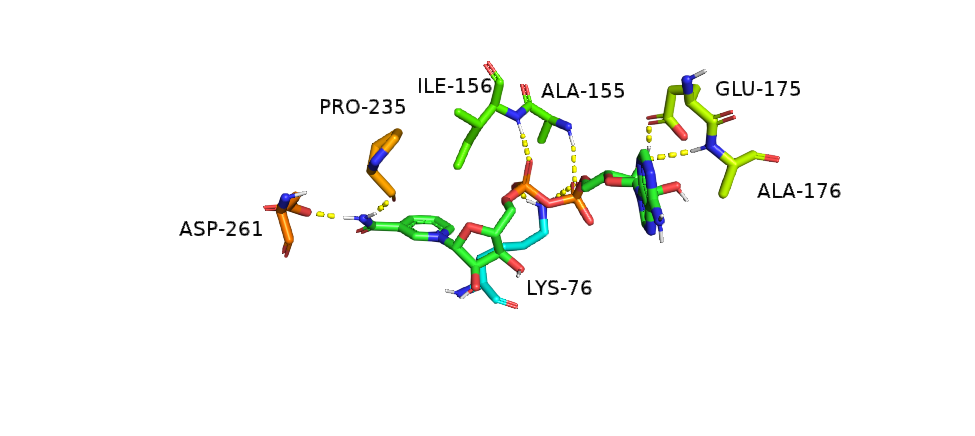 | **K177H**  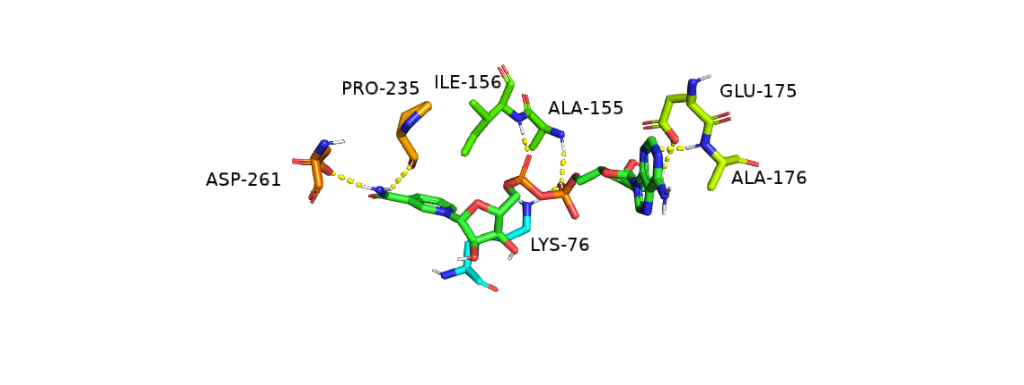 |
| **K177R**  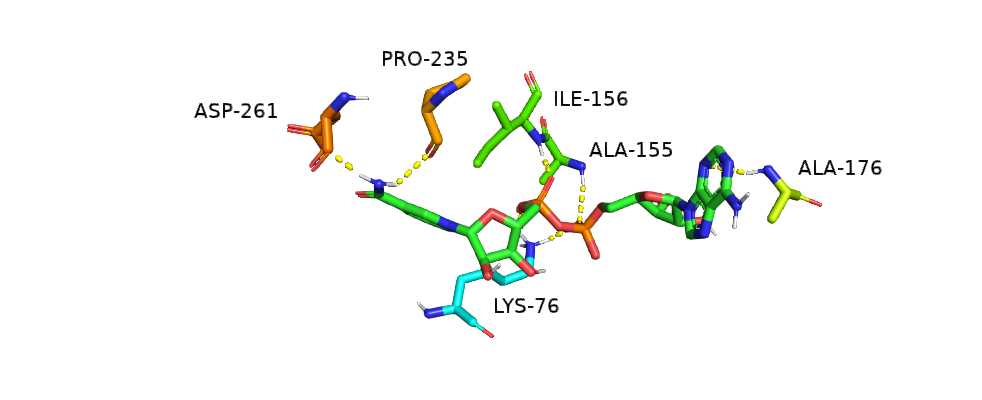 | **V262A**  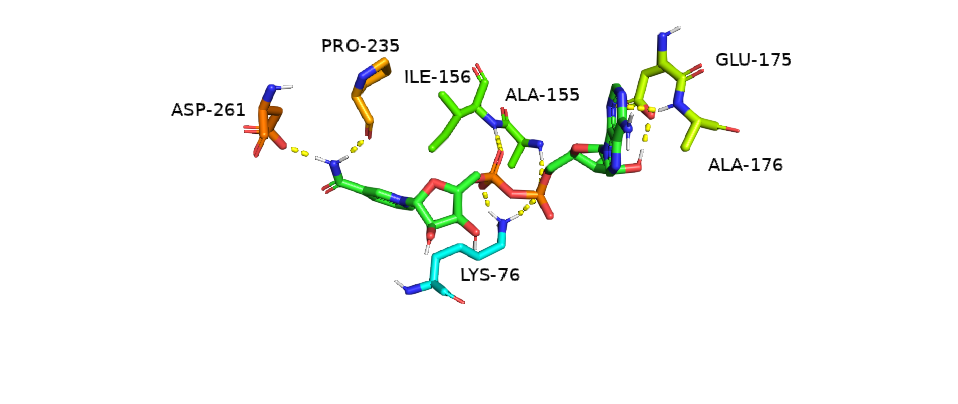 |
| **V262L**  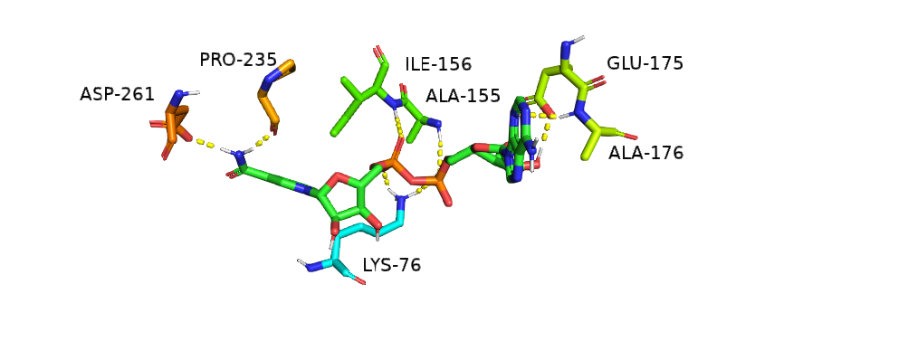 | **I293G**  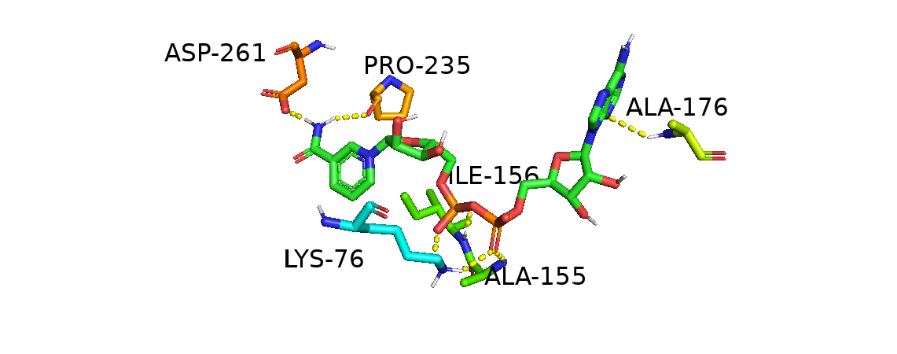 |
| **I293V**  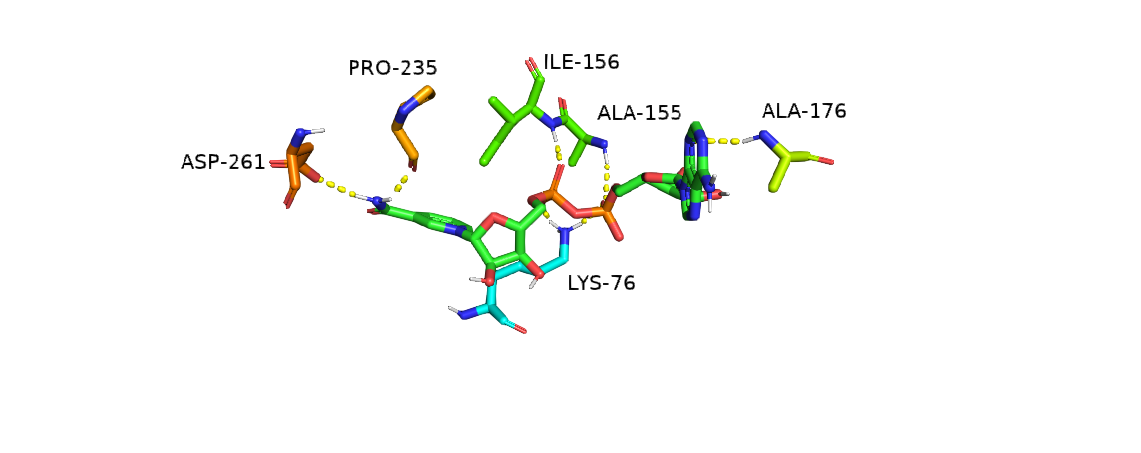 | **C236A**  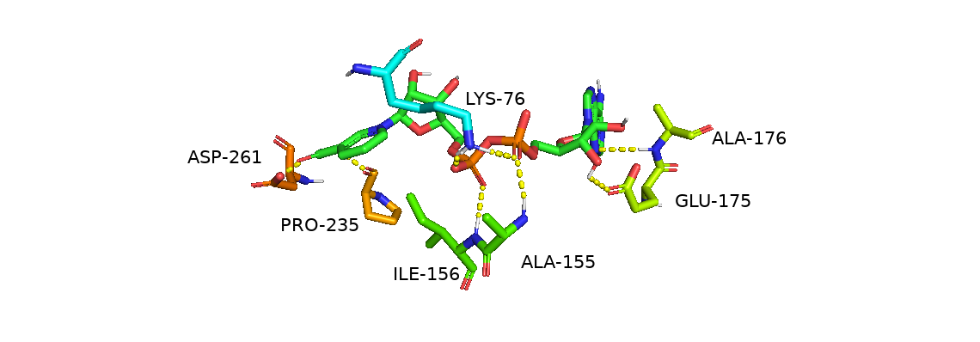 |
| **C236M**  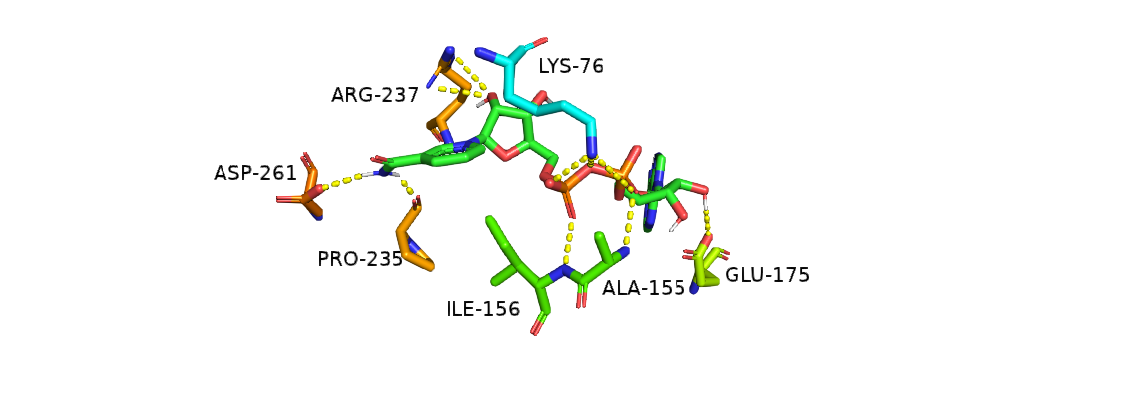 | **C236S**  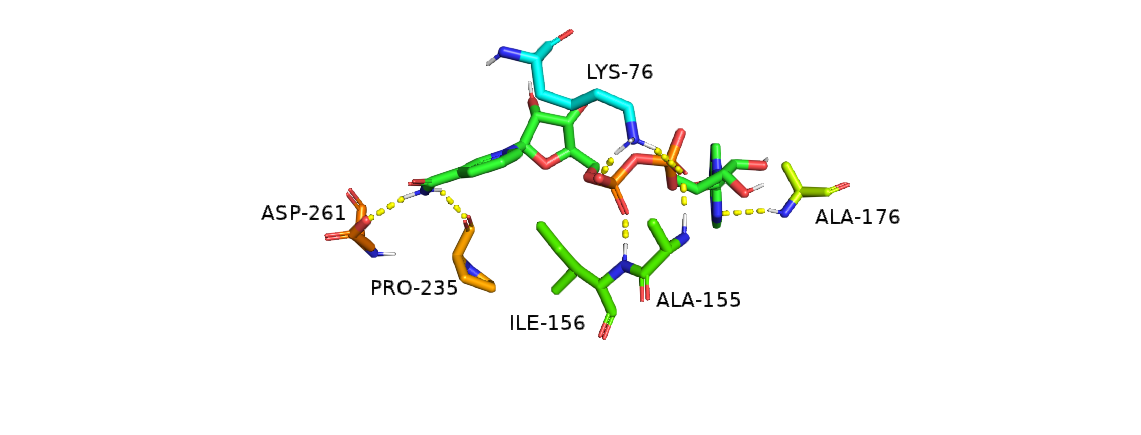 |
| **C236T**  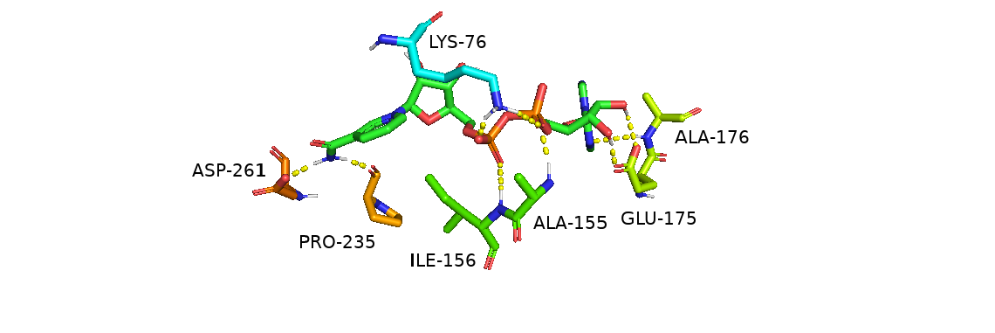 | **G157A**  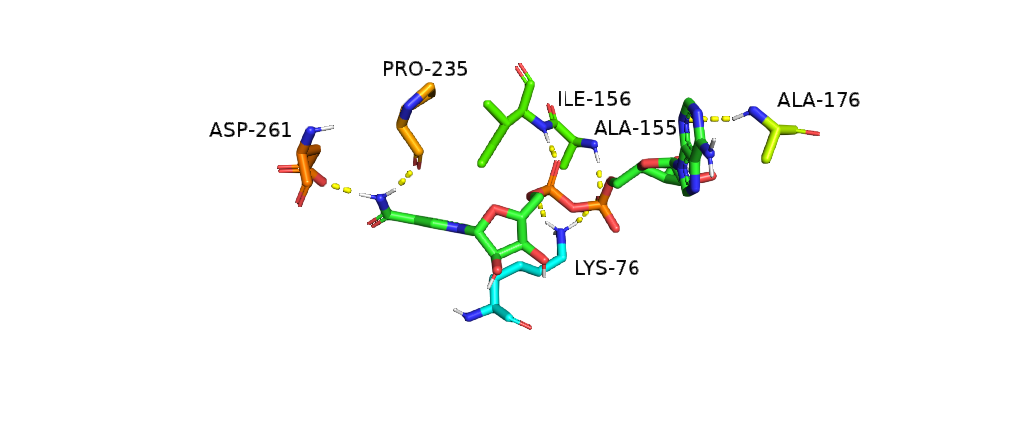 |
| **N211A**  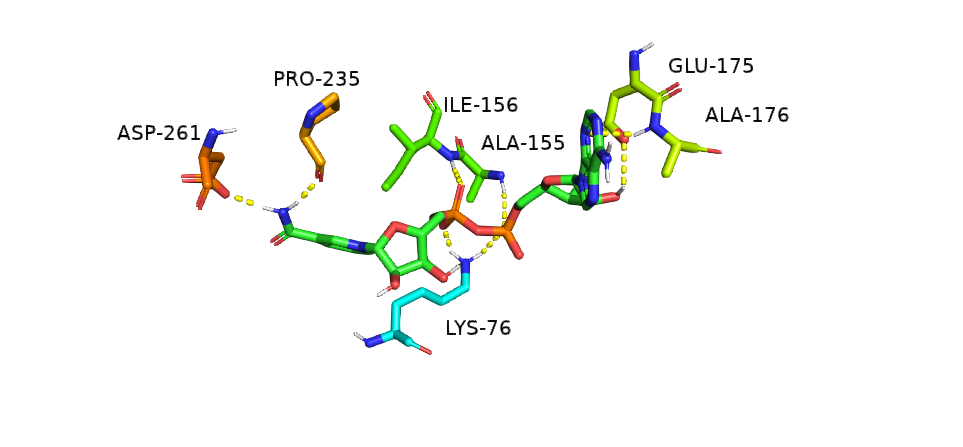 | **N211D**  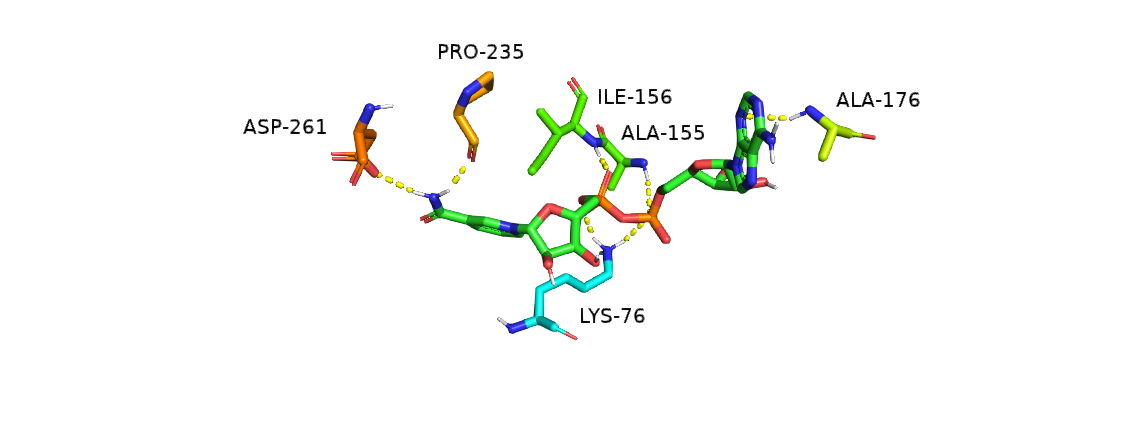 |

Figure S1: Docked pose of WT and mutants PTDH-NAD^+^ with lower binding affinity.

| **A155**  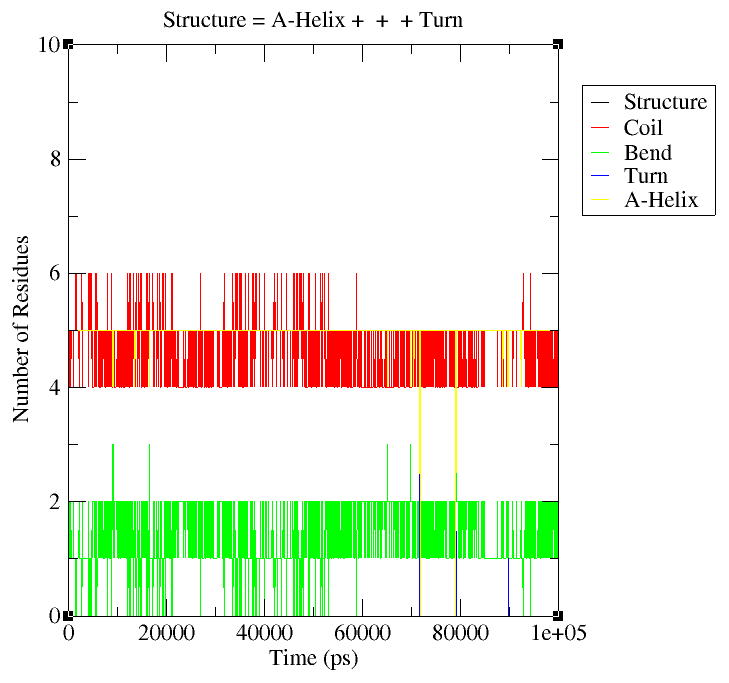 | **A155I**  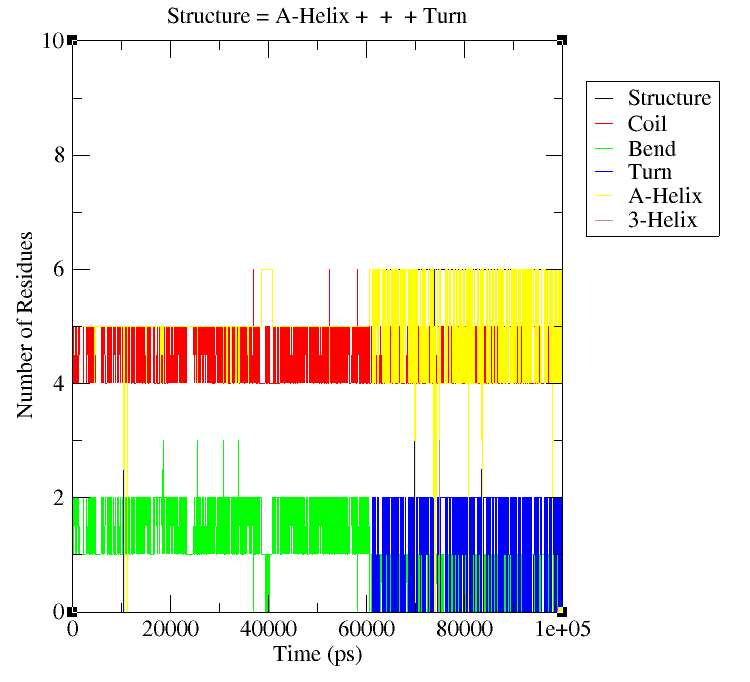 |
| --- | --- |
| **G157**  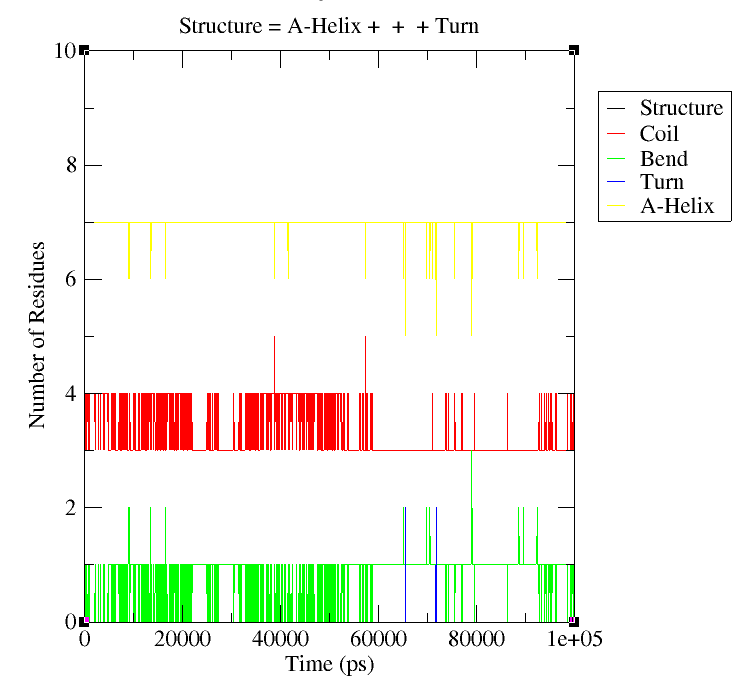 | **G157I**  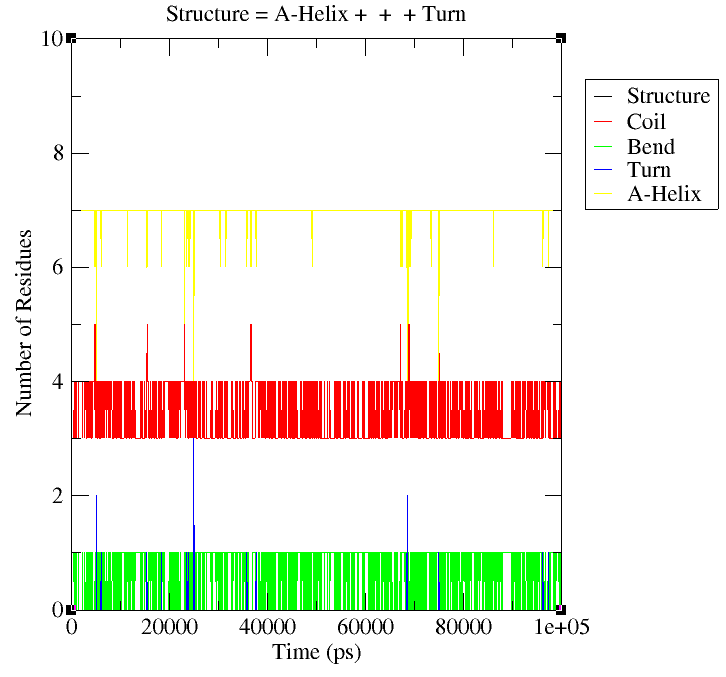 |
| **L217**  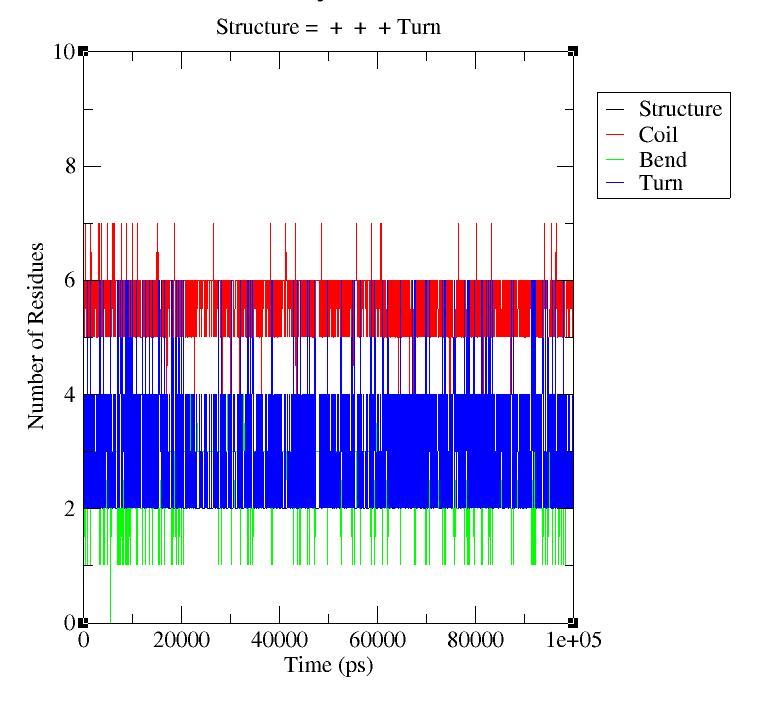 | **L217I**  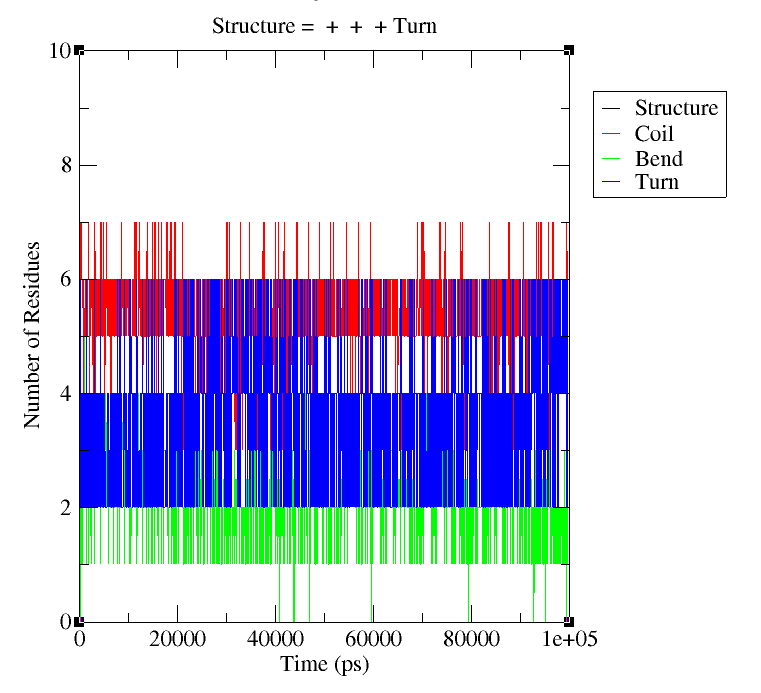 |
| **P235**  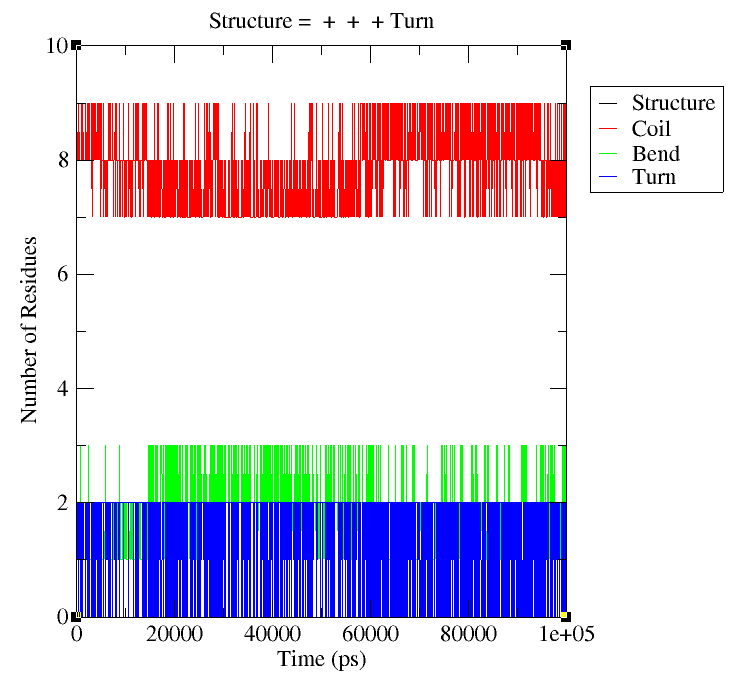 | **P235A**  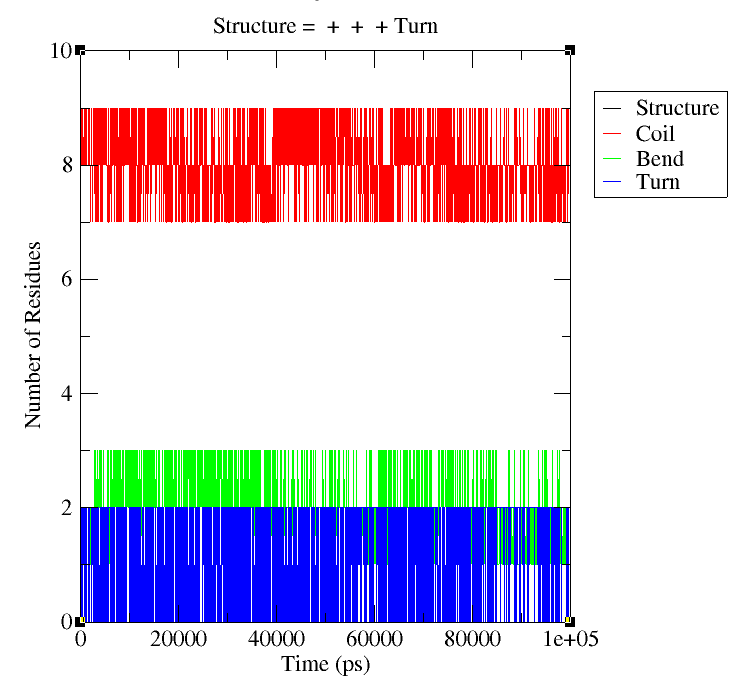 |
| **V262**  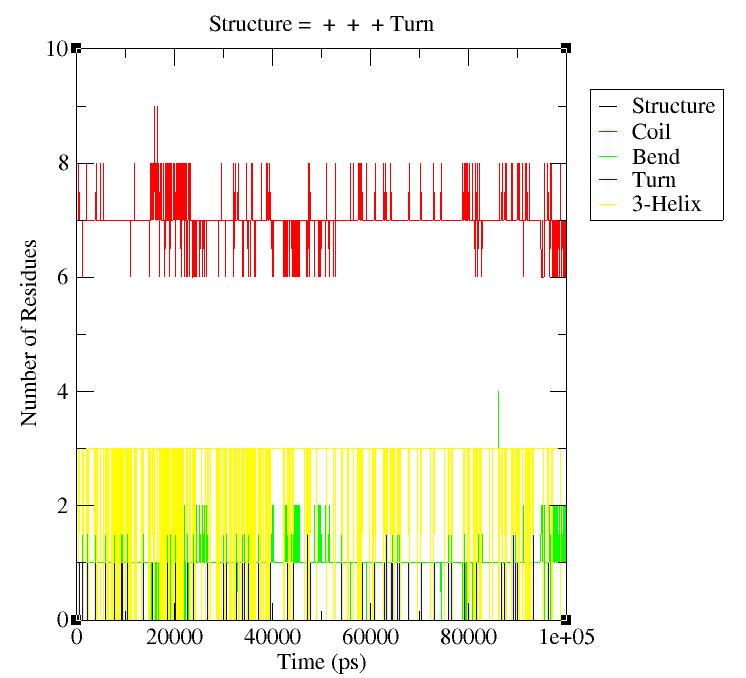 | **V262I**  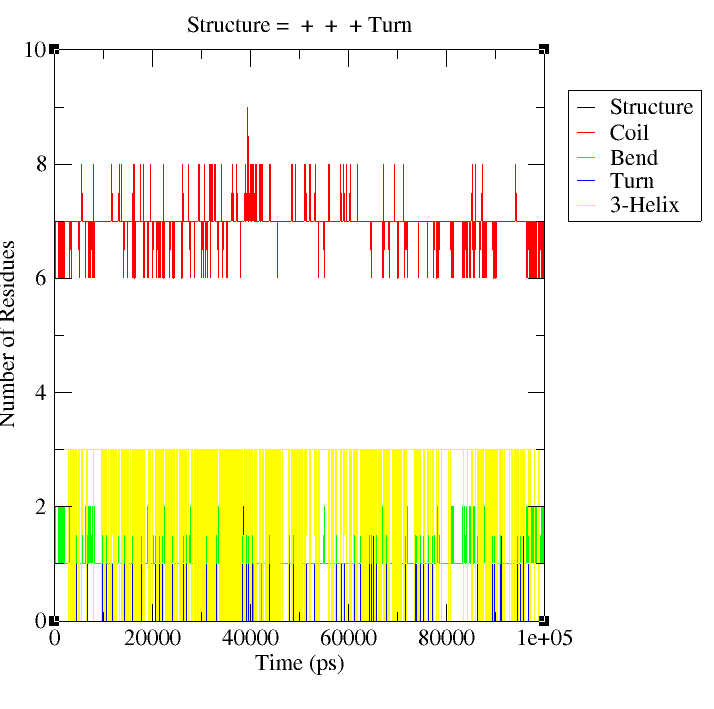 |
| **I293**  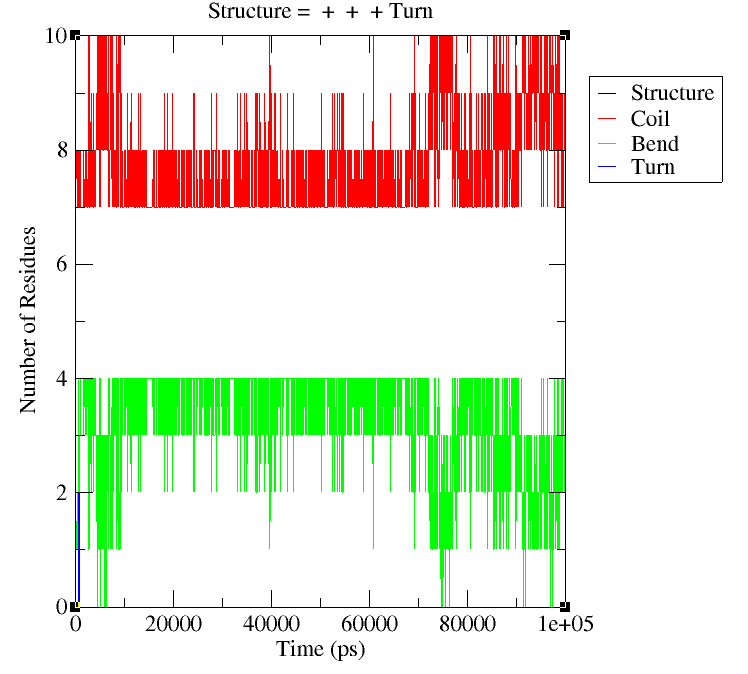 | **I293A**  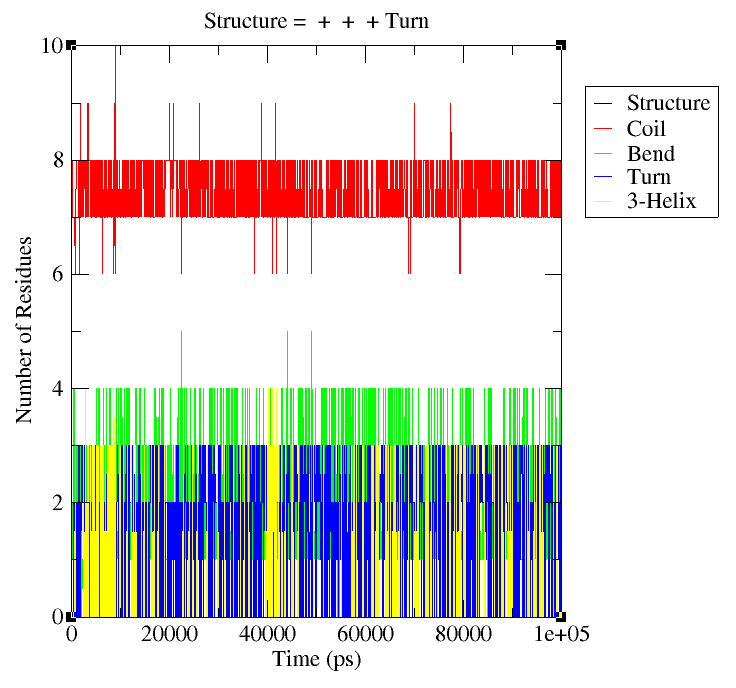 |
| **I293**  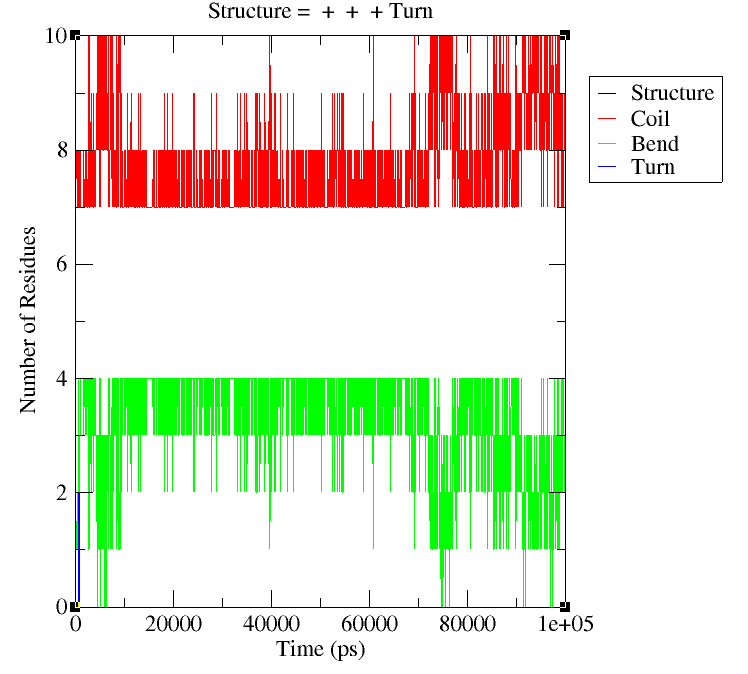 | **I293L**  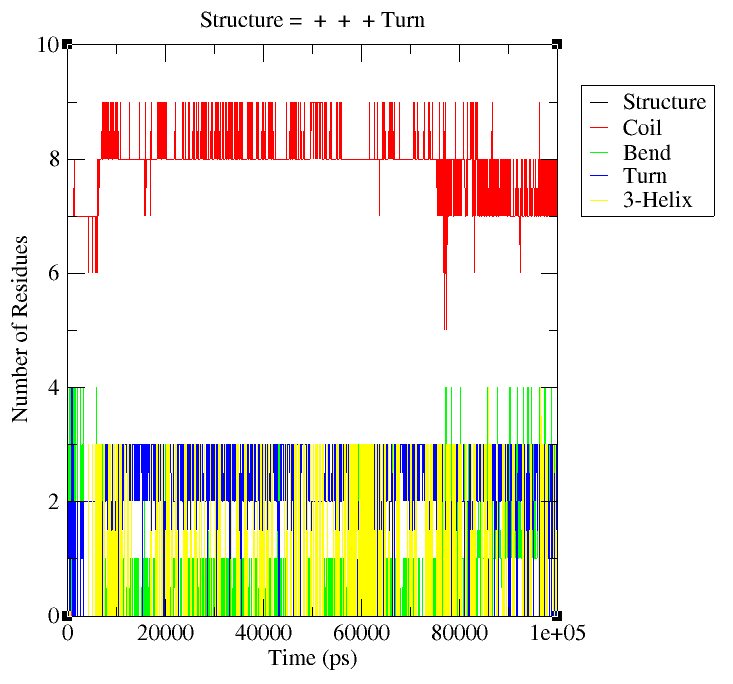 |

Figure S2: Secondary structure diagrams for PTDH protein WT and mutants.

Figure SD3: Graph plot showing calculated binding energy (kJ/mol) contributed by per residues of WT and selected mutants during interaction with NAD^+^.
